# Genome-Wide Characterization and Expression Profiling of the *AUXIN RESPONSE FACTOR* (*ARF*) Gene Family in *Eucalyptus grandis*

**DOI:** 10.1371/journal.pone.0108906

**Published:** 2014-09-30

**Authors:** Hong Yu, Marçal Soler, Isabelle Mila, Hélène San Clemente, Bruno Savelli, Christophe Dunand, Jorge A. P. Paiva, Alexander A. Myburg, Mondher Bouzayen, Jacqueline Grima-Pettenati, Hua Cassan-Wang

**Affiliations:** 1 LRSV Laboratoire de Recherche en Sciences Végétales, UMR5546, Université Toulouse III/CNRS, Castanet Tolosan, France; 2 Université de Toulouse, Institut National Polytechnique-Ecole Nationale Supérieure Agronomique de Toulouse, Laboratoire de Génomique et Biotechnologie des Fruits, Castanet-Tolosan, France; 3 Instituto de Investigação Científica e Tropical (IICT/MCTES), Lisboa, Portugal; 4 Instituto de Biologia Experimental e Tecnológica (IBET), Oeiras, Portugal; 5 Department of Genetics, Forestry and Agricultural Biotechnology Institute (FABI), Genomics Research Institute (GRI), University of Pretoria, Pretoria, South Africa; Leibniz Institute of Plant Biochemistry, Germany

## Abstract

Auxin is a central hormone involved in a wide range of developmental processes including the specification of vascular stem cells. Auxin Response Factors (ARF) are important actors of the auxin signalling pathway, regulating the transcription of auxin-responsive genes through direct binding to their promoters. The recent availability of the *Eucalyptus grandis* genome sequence allowed us to examine the characteristics and evolutionary history of this gene family in a woody plant of high economic importance. With 17 members, the *E. grandis ARF* gene family is slightly contracted, as compared to those of most angiosperms studied hitherto, lacking traces of duplication events. *In silico* analysis of alternative transcripts and gene truncation suggested that these two mechanisms were preeminent in shaping the functional diversity of the *ARF* family in *Eucalyptus*. Comparative phylogenetic analyses with genomes of other taxonomic lineages revealed the presence of a new ARF clade found preferentially in woody and/or perennial plants. High-throughput expression profiling among different organs and tissues and in response to environmental cues highlighted genes expressed in vascular cambium and/or developing xylem, responding dynamically to various environmental stimuli. Finally, this study allowed identification of three *ARF* candidates potentially involved in the auxin-regulated transcriptional program underlying wood formation.

## Introduction

The plant hormone auxin plays a prominent role in the regulation of plant growth in response to diverse developmental and environmental cues such as organogenesis, tropic movement, root growth, fruit development, tissue and organ patterning and vascular development [1]. Auxin plays a crucial role in the specification of vascular stem cells (procambium) and in cambial activity [2]. Analysis of auxin distribution across the cambial region in hybrid aspen trees showed a radial auxin gradient reaching a peak level in the cambial zone or at the border between the cambial zone and the expansion zone towards developing wood cells [3], [4]. The auxin gradient was indeed shown to overlap with the sequential and numerous auxin-regulated genes responding dynamically to the change in auxin levels in wood forming cells [5].

As trees are long living organisms with sessile lifestyle, they have to adapt to changing environmental conditions throughout their lifetimes which may span decades and centuries in some cases. In particular, vascular stem cell activity shows plasticity in response to mechanical stress which affects wood formation and quality. In angiosperm woody species, a local increase in cambial cell division induces the formation of tension wood in the upper side of the leaning tree stems [6], [7]. Auxin has been proposed to be implicated in the tension response, and application of either exogenous auxin or auxin transport inhibitors was shown to induce the gelatinous G-fibres characteristics of tension wood [8]. Although measurements of endogenous auxin failed to reveal significant changes in auxin balance in the cambial region tissues, a rather large set of auxin-related genes were found to be differentially expressed in developing poplar tension wood [9]. A recent study indicated that the auxin signalling pathway is significantly disrupted during cambial dormancy in hybrid aspen [10]. Despite the fact that auxin has long been proposed as primary regulator of cambial activity and wood formation [4], [11], the auxin-regulated transcriptional programs underlying wood formation remain largely under investigated.

Auxin exerts its function through modulating the expression of numerous genes among which is a set of transcriptional regulators. Auxin Response Factors (ARFs) and Aux/IAAs are two well-known mediators which regulate auxin responsive gene expression [12], [13]. Most ARF proteins contain a highly conserved N-terminal B3-like DNA binding domain that recognizes an auxin-response element (AuxRE: TGTCTC) present in the promoters of auxin-responsive genes. The C-terminal domain contains two motifs, called III and IV, also found in Aux/IAA proteins and shown to enable the formation of homo- and heterodimers among ARFs and Aux/IAAs [14], [15]. The middle region whose sequence is less conserved confers transcription activation or repression depending on its amino acid composition [13]. Biochemical and genetic studies in *Arabidopsis* and other plants have led to a working model of the mediation of auxin response by ARF proteins [14], [16]. In the absence of auxin, Aux/IAAs bind to ARFs and recruit co-repressors of the TOPLESS (TPL) family, preventing the ARFs from regulating target genes [17]. The presence of auxin induces Aux/IAA protein degradation via the 26S proteasome through SCF-TIR1 ubiquitin ligase complex; thus liberating the trapped ARF proteins, allowing them to modulate the transcription of target auxin-responsive genes (for review, see Guilfoyle and Hagen) [12]. This model based on limited ARF-Aux/IAA interaction studies which provides a framework for understanding how members of these families may function. More recently, a large-scale analysis of the Aux/IAA-ARF interactions in the shoot apex of *Arabidopsis* showed that the vast majority of Aux/IAAs interact with all ARF activators, suggesting that most Aux/IAAs may repress the transcriptional activity of ARF activators [18]. In contrast, Aux/IAAs have limited interactions with ARF repressors suggesting that the role of the latter is essentially auxin-independent and that they might simply compete with the ARF activators for binding to the promoter of auxin-inducible genes [18]. This finding is particularly important taking into account that auxin predominantly activates transcription [19]–[21] and that a large complement of the ARF family acts as transcriptional repressors [12]. Whereas the above proposed scenario applies to the shoot apical meristem, it is likely that specific interactions between Aux/IAAs and ARFs might also affect the dynamics of the ARF-Aux/IAA signalling pathway in other developmental processes such as cambial development.

The *ARF* gene family has been most extensively studied in *Arabidopsis* where phenotyping of mutants revealed involvement of specific *ARF* genes in various plant growth and development processes [20], [22]–[30]. For instance, ARF5/MONOPTEROS (MP) is a transcriptional activator known to play a critical role in the specification of vascular stem cells [27], [31].

The *ARF* family has also been characterized in several annual herbaceous plants including monocots (rice, maize) [32], [33] and dicots (*Arabidopsis*, tomato, soybean, *Brassica rapa*) [24], [34]–[37] and in only two woody perennial genera, *Populus* [38] and *Vitis* [39]. However, so far, no *ARF* candidate has been identified as specifically involved in vascular cambium activity and xylem differentiation.

The recent availability of *Eucalyptus grandis* genome [40], the second hardwood forest tree genome fully sequenced, offers new opportunities to get insights into the regulation of secondary growth and cambial activity by ARFs, especially because *Eucalyptus* belongs to evergreen trees that do not present dormancy in their cambial activity in sharp contrast with deciduous trees like *Populus*. *Eucalyptus* is also the most planted hardwood in the world, mainly for pulp and paper production but is also foreseen as a dedicated energy crop for lignocellulosic biofuel production. Thus, understanding the mechanisms that underlying auxin regulation in *Eucalyptus* wood formation is of interest both in the context of plant development and as a path to improve lignocellulosic biomass production and quality.

In the present paper, we report a genome-wide identification and characterization of the *ARF* family in *Eucalyptus grandis*. We analyzed gene structure, protein motif architecture, and chromosomal location of the members of the *E. grandis ARF* family. We also performed comparative phylogenetic relationships and large scale transcript profiling with a special focus on vascular tissues to get insights in their evolution, expression characteristics and possible functions.

## Materials and Methods

### Identification of *ARF* gene family in *Eucalyptus grandis* and chromosomal location

The identification procedure is illustrated in Fig. S1. Firstly we used *Arabidopsis* ARF proteins as queries in BLASTP searches for predicted protein in *Eucalyptus* genome (JGI assembly v1.0, annotation v1.1, http://www.phytozome.net/eucalyptus). A total of 64 *Eucalyptus* proteins identified in this initial search were examined by manual curation of protein motif scan using Pfam domain IDs (http://pfam.wustl.edu) and NCBI conserved domain database (http://www.ncbi.nlm.nih.gov/cdd). Redundant and invalid gene models were eliminated based on gene structure, intactness of conserved motifs and EST support. Three incomplete gene models were identified and completed by FGeneSH (http://linux1.softberry.com). To complete partial sequence of Eucgr.K02197.1, we cloned the corresponding genomic fragment using forward primer: 5′-AATTGACCGCGGTTGGATA-3′ and reverse primer 5′-GAGCAGGCCAACATCCTCA-3′, which located up-stream and down-stream respectively of the non-determined sequence (N). According to sequencing result we complete the missing part (1156 bp), corresponding to a part of promoter region and a part of 5′end CDS of the Eucgr.K02197.1 (submitted to GenBank data library under the accession number KC480258). All these manual curations enabled us to obtain 17 complete *Eucalyptus* ARF proteins sequences. We then used them as query in two subsequent additional searches: 1) BLASTP against *Eucalyptus* proteome for exhaustive identification of divergent *Eucalyptus* gene family members, and 2) tBLASTn searches against *Eucalyptus* genome for seeking any possible non-predicted genes. For validation, we also used poplar ARF proteins as queries to do the search procedure described above, and we obtained exactly the same result.

In the course of the above identification process we completed and expertly re-annotated three partial sequences (accession numbers Eucgr.F02090.1, Eucgr.F04380.1, and Eucgr.K03433.1 in the Phytozome database) initially annotated in the *Eucalyptus* genome-sequencing project (Table 1). In addition, we found one gene (accession number Eucgr.K02197.1) that corresponded to a partial sequence for which the 5′ end was not determined (1240 N as sequencing results). Information on chromosomal location was retrieved from the *Eucalyptus* genome browser (http://www.phytozome.net/eucalyptus). *EgrARF* genes were mapped to their loci using MapChart 2.2 [41].

**Table 1:** *ARF* gene family in *Eucalyptus*.

Accession no.^a^	Arabidopsis best hit (blastp)^b^	Amino acid identity %^c^	Short name^d^	Number of predicted alternative transcript^e^	Chromosome^f^	Genome location^g^	ORF (bp)^h^	Deduced polypetide^i^			Exon No.
								Length (aa)	MW (kDa)	PI	
*Eucgr.G00076.1*	*AtARF1*	62.2	*EgrARF1*	4	7	848,248..860,087	2028	675	75.3	6.06	14
*Eucgr.K02197.1*	*AtARF2*	59.3	*EgrARF2A* **	5	11	29,232,770..29,239,103	2508	835	93.13	6.55	14
*Eucgr.B03551.1*	*AtARF2*	49.5	*EgrARF2B*	2	2	60,136,697..60,143,120	2364	787	88.09	6.62	14
*Eucgr.D00588.1*	*AtARF3*	46.2	*EgrARF3*	2	4	10,835,264..10,841,977	2172	723	79.19	6.36	10
*Eucgr.B02480.1*	*AtARF4*	57.8	*EgrARF4*	3	2	46,950,723..46,956,747	2394	797	88.53	6.31	12
*Eucgr.F02090.1*	*AtARF5*	59.3	*EgrARF5* *	1	6	28,226,789..28,233,884	2835	944	103.53	5.43	14
*Eucgr.D00264.1*	*AtARF6*	69.1	*EgrARF6A*	2	4	4,224,849..4,232,295	2694	897	99.04	6.04	14
*Eucgr.A02065.1*	*AtARF6*	65.6	*EgrARF6B*	3	1	31,463,716..31,473,722	2613	870	96.64	6.06	14
*Eucgr.D01764.1*	*AtARF9*	58.6	*EgrARF9A*	1	4	31,589,333..31,593,948	1971	656	73.53	6.15	14
*Eucgr.E00888.1*	*AtARF9*	58.4	*EgrARF9B*	2	5	9,303,573..9,308,209	2064	687	76.46	6.09	14
*Eucgr.J00923.1*	*AtARF10*	60.1	*EgrARF10*	1	10	10,070,677..10,074,840	2139	712	77.68	8.32	4
*Eucgr.G02838.1*	*AtARF16*	58	*EgrARF16A*	1	7	46,569,603..46,573,031	2079	692	76.33	6.80	3
*Eucgr.K01240.1*	*AtARF16*	52.5	*EgrARF16B*	1	11	15,584,770..15,588,716	2124	707	77.91	6.91	3
*Eucgr.F04380.1*	*AtARF17*	42.1	*EgrARF17* *	1	6	52,730,940..52,733,856	1881	626	67.75	7.54	3
*Eucgr.C03293.1*	*AtARF19*	60.4	*EgrARF19A*	2	3	62,460,408..62,469,132	3360	1119	124.93	6.30	14
*Eucgr.C02178.1*	*AtARF19*	44.8	*EgrARF19B*	2	3	39,505,080..39,513,066	3360	1119	123.24	5.98	14
*Eucgr.K03433.1*	*-*	-	*EgrARF24* *	1	11	43,352,151..43,357,873	1836	611	68.44	7.24	14

*Using FGeneSH to complete the complete sequence.

**using specific primers to amplify the genomic DNA to complete the sequence.

a Gene model of Eucalyptus (version 1.1) in phytozome V8.0.

b The best hit of EgrARF in Arabidopsis by using blastp.

c The amino acid identity percentage between EgrARF and corresponding AtARF.

d Designation related to Arabidopsis best hit.

e The number of predicted alternative transcripts of EgrARF in phytozome.

f,g Location of the EgrARF genes in the Chromosome.

h Length of open reading frame in base pairs.

i The number of amino acids, molecular weight (kilodaltons), and isoelectric point (pI) of the deduced polypeptides.

### Sequence, phylogenetic, gene structure analysis

Conserved protein motifs were determined by Pfam [42]. Multiple protein sequences alignment was performed using Clustal X program (Version 2.0.11). Using full length sequences of all predicted protein, phylogenetic trees were constructed with MEGA5 program by neighbor-joining method with 1000 bootstrap replicates. Their exon-intron structures were extracted from Phytozome (http://www.phytozome.net/eucalyptus) and visualized in Fancy Gene V1.4 (http://bio.ieo.eu/fancygene/). The prediction of small RNA target sites in *EgrARF* genes was performed through the web application psRNATarget (http://plantgrn.noble.org/psRNATarget/). The stem-loop structures were predicted using RNAfold web server (http://rna.tbi.univie.ac.at/cgi-bin/RNAfold.cgi) and visualized by RNAstructure 5.3 program.

### Plant material

The plant materials provenance and preparation are described in Cassan-Wang et al. [43]. Hormone treatments were performed in an *in vitro* culture system. 10 µM NAA (1-Naphthaleneacetic acid, for auxin), or 20 µM gibberellic acid or 100 µM ACC (1-aminocyclopropane-1-carboxylic-acid, for ethylene) were added to the medium of 65-d-old young trees, and trees were sampled 14 days after treatments.

### Total RNA extraction, cDNA synthesis, quality controls and high throughput quantitative RT-PCR

All the procedures used for the qRT-PCR, from the RNA extraction to the calculation of transcript abundance are described in Cassan-Wang et al. [43]. Only samples with a RNA integrity number >7 (assessed by Agilent 2100 Bioanalyzer) were retained for reverse transcription. cDNA quality was assessed as described by Udvardi et al. [44] using housekeeping genes *IDH* and *PP2A3* (primers see Table S1). Primer pairs were designed using the software QuantPrime (http://www.quantprime.de) [45], showing in Table S1. qRT-PCR was performed by the Genotoul service in Toulouse (http://genomique.genotoul.fr/) using the BioMark 96∶96 Dynamic Array integrated fluidic circuits (Fluidigm Corporation, San Francisco, USA) described in Cassan-Wang et al. [43]. The specificity of the PCR products was confirmed by analysing melting curves. Only primers that produced a linear amplification and qPCR products with a single-peak melting curves were used for further analysis. The efficiency of each pair of primers was determined from the data of amplification Ct value plot with a serial dilution of mixture cDNA and the equation E = 10^(-1/slope)^ -1. E^-ΔΔCt^ method was used to calculate relative mRNA fold change compared to control sample using formula (*E*_target_)^ΔCt_target (control−sample)^/(*E*_reference_)^ΔCt_reference (control−sample)^ [46] and five reference genes (*IDH, PP2A1, PP2A3, EF-1a* and *SAND*, Table S1) were used for data normalization. We chose *in vitro* plantlets as control sample, because it contains the main organs and tissues of our studies such as stem, leaves, shoot tips, xylem, phloem and cambium, and it is a relative stable and less variable sample as being grown under the same *in vitro* culture condition from one experiment to another.

### Transactivation analysis in single cell system

For testing the ability of ARF transcription factors to up or down regulate the expression of auxin responsive promoter DR5, the full-length cDNAs of the ARF transcription factors were cloned in pGreen vector under 35SCaMV promoter to create the effector constructs. The reporter constructs use a synthetic auxin-responsive promoter DR5 fused with the GFP reporter gene. Tobacco BY-2 protoplasts were co-transfected with the reporter and effector constructs as described in Audran-Delalande et al. [47]. After 16 h incubation, GFP expression was quantified by flow cytometry (LSR Fortessa, BD Biosciences). Data were analysed using BD FacsDiva software. Transfection assays were performed in three independent replicates and 3000–4000 protoplasts were gated for each sample. GFP fluorescence corresponds to the average fluorescence intensity of the protoplasts population after subtraction of auto-fluorescence determined with non-transformed protoplasts. 50 µM 2, 4-D was used for auxin treatment. We tested two independent protoplast preparations and for each of them, we performed in three independent transformation replicates. Similar results were obtained with the independent protoplast preparations and the data were represented by one of the preparations. For normalization, protoplasts were transformed with the reporter vector and the effector plasmid lacking the *ARF* gene.

## Results and Discussion

### Identification and chromosomal distribution of *Eucalyptus ARF* genes

The procedure to identify all members of the *ARF* family in the *E. grandis* genome (JGI assembly v1.0, annotation v1.1 (http://www.phytozome.net/cgi-bin/gbrowse/eucalyptus/), included expert manual curation as illustrated in Fig. S1. It allowed the identification of 17 genes encoding full length *Eucalyptus* ARF proteins (henceforth referred to as *EgrARF* genes). We named these genes according to their potential orthologs in *Arabidopsis* (Table 1). Where two *EgrARFs* matched the same potential *Arabidopsis* ortholog *AtARFx*, they were named as *EgrARFxA* and *xB*, with *xA* being the closest to the *Arabidopsis* ortholog; e.g. *EgrARF2A* and *EgrARF2B*. The percentage of identity between the *Arabidopsis* and the *Eucalyptus* predicted ARF protein sequences, and among the *Eucalyptus* ARFs themselves are given as Table S2 and S3, respectively. Eight *Arabidopsis* genes have no corresponding *Eucalyptus* orthologs (*AtARF12* to *15* & *20* to *23*), while only one *EgrARF* gene, *EgrARF24*, has no ortholog in *Arabidopsis* (Table 1). *In silico* chromosomal mapping of the gene loci revealed that the 17 *EgrARF* genes are scattered on nine of the eleven chromosomes, with one to three *EgrARF* genes per chromosome and with chromosomes 8 and 9 being devoid of *ARF* genes (Fig. S2).

**Figure 1:**
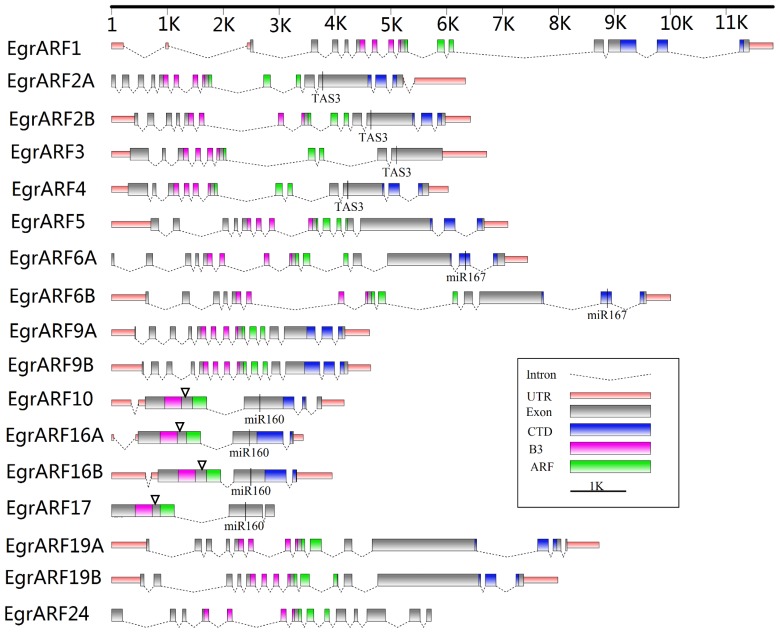
Gene structure of the *EgrARF* family. The information on exon–intron structure was extracted from the Phytozome database and visualized by using the FancyGene software (http://bio.ieo.eu/fancygene/). The sizes of exons and introns are indicated by the scale at the top. The domains of *EgrARF* gene were predicted by Pfam (http://pfam.xfam.org/) and are indicated by different colours. The B3 together with ARF subdomains constitute the DNA binding domain (DBD). The CTD contains two sub-domains III and IV. The TAS3 and microRNA target sites are marked on the corresponding target genes. The triangles underline the insertion sites of additional short amino-acids segments between the B3 and ARF subdomains.

The predicted proteins encoded by the *EgrARF* genes ranged from 593–1119 amino acid residues (Table 1), with PIs in the range of 5.43–8.32, suggesting that they can work in very different subcellular environments. Sequence analyses of the predicted proteins and Pfam protein motif analysis showed that most of them (14 of the 17 predicted proteins) harbour the typical ARF protein structure comprising a highly conserved DNA-binding domain (DBD) in the N-terminal region composed of a plant specific B3-type subdomain and an ARF subdomain, a variable middle region (MR) that functions as an activation or repression domain, and a carboxy-terminal dimerization (CTD) domain consisting of two highly conserved dimerization subdomains III and IV, similar to those found in Aux/IAAs (Fig. 1). We analysed and aligned the predicted amino acid sequences of the *Egr*ARFs (Fig. 1 and Fig. S3). Four out of the 17 *Egr*ARFs (*10, 16A, 16B* and *17*) exhibited an additional short segment of amino acids (between 15 to 43 amino-acids) in their DBD, between the B3 and ARF subdomains (Fig. 1 and Fig. S3). Such a feature has already been reported in *Arabidopsis* and soybean [36]. At the end of the DBD domain, all of the *Egr*ARFs excepty *Egr*ARF6A, 6B and 19A contain a conserved putative mono-partite nuclear localization signal (NLS) (Fig. S3) shown to direct the proteins into the nucleus [36], [48].

Five *Egr*ARFs (*Egr*ARF5, 6A, 6B, 19A and 19B) harbour a glutamine (Q)-rich middle region (Fig. S3) implying that these proteins are likely transcriptional activators since glutamine enrichment seems to be a distinctive feature of ARF activators in all plant lineages [15], [49]. The other 12 *Egr*ARFs may function as repressors based on their middle regions enriched either in S (serine), SPL (Serine, Proline, Leucine) or SGL (Serine, Glycine, Leucine) [36] (Fig. S3).

The predicted protein structures of *Egr*ARF3 and *Egr*ARF17 are lacking dimerization domains III and IV like their potential orthologs in *Arabidopsis* (Fig. 1 and Fig. S3). *Egr*ARF24, which has no ortholog in *Arabidopsis*, has a truncated CTD since only Aux/IAA subdomain III is present. The percentage of *Egr*ARFs displaying a truncated CTD (17.6%) is similar to that in *Arabidopsis* (17.4%), but lower than in rapeseed (22.6%) or tomato (28.6%) [24], [37], [50]. These truncated *Egr*ARFs are predicted to be unable to interact with Aux/IAA, a sequestration mechanism which may regulate their activity, and hence, they are likely to be insensitive to auxin. However, ARF repressors seem to display very limited interactions with Aux/IAA proteins [18], therefore the lack of domains III and/or IV could also have consequences for the interaction of ARFs with other transcriptional regulators [49].

Compared to *Arabidopsis*, the ARF family in *Eucalyptus* is slightly contracted with 17 *versus* 23 members. It is worth noting that we found the exact same number of *ARF* genes in another *Eucalyptus* species, *E. camaldulensis* (http://www.kazusa.or.jp/eucaly/). Indeed when comparing to other species, in which the *ARF* family has been characterized (Table 2), *Eucalyptus* and grapevine appeared to have the smallest families with 17 and 19 members respectively, whereas poplar and soybean had the largest families with 39 and 51 members, respectively. We did not find evidence that any of the 17 *EgrARF* genes arose by tandem, segmental, or whole genome duplication, or even the more ancient hexaploidization in the *E. grandis* genome [40] and it appears that any such duplicates have been lost in *Eucalyptus* as is the case for 95% of whole-genome duplicates. This is sharply contrasting with the intensive tandem duplication events found for *Arabidopsis* ARF members [14], [51], the segmental duplication found in *Populus* [38], and the whole-genome duplication events in soybean [36].

**Table 2:** Summary of *ARF* gene content in angiosperm species.

Species	ARFs content	Reference
*Eucalyptus grandis*	17	This study
*Vitis vinifera*	19	[39] Wan *et al.* (2014)
*Solanum lycopersicon*	22	[35] Zouine *et al.* (2014)
*Arabidopsis thaliana*	23	[24] Okushima *et al*. (2005)
*Oryza sativa*	25	[33] Wang *et al.* (2007)
*Zea mays*	31	[32] Xing *et al.* (2011)
*Brassica rapa*	31	[37] Mun *et al*. (2012)
*Populus trichocarpa*	39	[38] Kalluri *et al*. (2007)
*Glycine max*	51	[36] Ha *et al.* (2013)

As duplication and alternative splicing are the two main mechanisms involved in diversification of function within gene families, sometimes viewed as opposite trends in gene family evolution, we performed an *in silico* survey of the alternative transcripts predicted in the *E. grandis* genome JGI assembly v1.0, annotation v1.1 (http://www.phytozome.net/eucalyptus), and compared them to those in *Arabidopsis* (Table 1 and Fig. S4). More than half of the *Eucalyptus* ARF family members (10 out of 17) have evidence of alternative splicing (Fig. S4). Taking into account the number of possible alternative transcripts in *Eucalyptus* (17) and in *Arabidopsis* (15), the total number of possible transcripts in both species becomes very similar, 34 and 38, respectively. Some of the transcripts resulted in truncated versions of the genes like *EgrARF1.4, 4.3* and *9B.2* lacking the Aux/IAA interaction domain and *EgrARF2B.2* lacking the B3/DBD domain. We further compared the *in silico* predicted *ARF* alternative transcripts from *E. grandis* to those expressed in a dataset of in-house RNA-Seq data from *E. globulus* (Table S4, Fig. S5, File S1). Remarkably, the vast majority of the alternative transcripts predicted in *E. grandis* were found expressed in *E. globulus* providing strong experimental support to their occurrence and conservation in the two *Eucalyptus* species. The importance of alternative splicing in the *ARF* family, has been highlighted recently by Finet et al. [49], who have shown that two *Arabidopsis* alternative transcripts of *AtARF4* have very different functions in flower development, and by Zouine et al. [35] who have shown that in tomato, one third of the *ARF* members displays alternative splicing as a mode of regulation during the flower to fruit transition. In *Arabidopsis* and in many other species, not only domain rearrangement through alternative splicing but also extensive gene duplication played a significant role in *ARF* functional diversification [49], whereas in *Eucalyptus* the first mechanism appeared to be preeminent.

### Comparative Phylogenetic analysis of the *ARF* family

**Figure 2:**
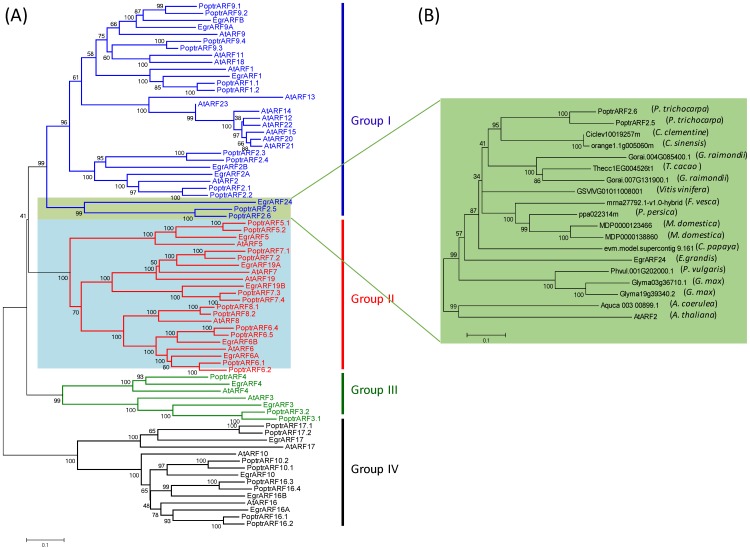
Phylogenetic relationships of ARF proteins between *Eucalyptus* and other species. (A) Phylogenetic relationships between ARF proteins from *Arabidopsis*, *Populus* and *Eucalyptus*. Full-length protein sequences were aligned by using the Clustal_X program. The phylogenetic tree was constructed by using the MEGA5 program and the neighbor-joining method with predicted ARF proteins. Bootstrap support is indicated at each node. The blue shade highlights the activators, and the green shade indicates the distinct likely woody preferential clade containing *EgrARF24*. (B) Phylogenetic relationships between the orthologs of *EgrARF24* in other species. *EgrARF24* proteins were used to blast 33 species genomes in Phytozome. An E-value of 1.0E-50 as used as a cut off to select the ARF potential orthologs from each species. A phylogenetic tree was constructed used the procedure as in (A) and using *AtARF2* was used as an outgroup. The species containing putative orthogs of *EgrARF24* were the followings: 1 *Aquilegia coerulea*, 2 *Glycine max*, 1 *Phaseolus vulgaris*, 1 *Carica papaya*, 2 *Malus domestica*, 1 *Prunus persica*, 1 *Fragaria vesca*, 1 *Vitis vinifera*, 2 *Populus trichocarpa*, 1 *Citrus sinensis*, 1 *Citrus clementine*, 2 *Gossypium raimondii*, 1 *Theobroma cacao*.

To assess the relationship of *Eucalyptus ARF* family members to their potential orthologs in other landmark genomes, we constructed a comparative phylogenetic tree using all predicted ARF protein sequences from genomes of relevant taxonomic lineages. The core rosids were represented by *Arabidopsis* and *Populus* (Malvids) while the Myrtales, the Vitales and the Asterides were represented by *Eucalyptus grandis*, *Vitis vinifera* and *Solanum lycopersicum*, respectively. The monocots were represented by the O*ryza sativa* genome (Fig. S6). A simplified tree with only *Arabidopsis*, *Populus* and *Eucalyptus* (Fig. 2A) showed that ARFs are distributed into four major groups I, II, III, and IV. *Eucalyptus* (and also grapevine) which harbour the smallest number of *ARF* genes as compared to all other species (Table 2), have the fewest number of ARF proteins in each of the four groups. The positions and phases of the introns were well conserved within each group (Fig. 1 and Fig. 2), whereas their sizes were poorly conserved even within the same group. All five predicted *Eucalyptus* ARF transcriptional activators fell within group II as their potential orthologs from *Arabidopsis* and other species; the remaining *Egr*ARFs were distributed among the three other groups.

Some lineage-specific clades were found in the *Solanaceae ARF* family [35] as well as in *Arabidopsis ARF* family [24]. In *Arabidopsis*, group I was substantially expanded with a subgroup containing seven tandem duplicated genes (encoding proteins AtARF12 to 15 and AtARF20, to 22), and the sister pair of AtARF11–AtARF18, for which orthologs were found only in Brassicaceae.

In group I, an isolated clade (highlighted in green in Fig. 2) contained *Egr*ARF24 clustering with *Ptr*ARF2.5 and *Ptr*ARF2.6 and did not contain any obvious *Arabidopsis* ortholog. This clade was absent from the herbaceous annual plants (*Arabidopsis*, tomato and rice), but present in woody perennial plants (*Eucalyptus*, *Populus* and *Vitis*; Fig. S6). To verify if this clade could be more specific to woody perennial plants, we performed a BLAST similarity search in 33 plant genomes available in Phytozome and found potential orthologs of *EgrARF24* in 13 plant species out of 33 (Table S5) which are presented in a phylogenetic tree (Fig. 2B). Among these 13 plant species, 11 are trees such as *M. domestica, C. sinensis, C. clementina, P. persica*, or tree-like plants and shrubs such as *C. papaya, T. cacao*, *G. raimondii*, although the latter is often grown as an annual plant. *A. coerulea and F. vesca* are perennial herbaceous plants. The two notable exceptions are two members of the Fabaceae family (*G. max*, and *P. vulgaris*) which are annual herbaceous plants. We thus considered this clade as woody-preferential. Regarding Group III, there was no evidence of large expansion of *ARF3* and *ARF4* genes in any of the three species, with only *ARF3* duplicated in *Populus*. Group IV contained four members from *Eucalyptus*, i.e. one more than in *Arabidopsis*. All of the *Egr*ARFs belonging to this group have in common an additional fragment (between 15 to 43 amino-acids residues) within their DBD (Fig. 1 and Fig. 2) and, noteworthy, alternative splicing was not detected for any of these genes in *Eucalyptus* and *Arabidopsis* (Fig. S4).

### Prediction of small regulatory RNAs and their potential ARF targets

In *Arabidopsis*, several *ARF* genes are targets of microRNAs miR160 and miR167, or of a trans-acting short interfering RNA (tasiRNA) *TAS3* [29], [52]–[54]. Since these small RNAs and their targets are very often conserved across plant species [32], [55], [56], we searched for their potential orthologs in the *Eucalyptus* genome. Their chromosomal locations, genomic sequences and the sequences of their mature forms are presented in Table S6. We identified three potential *Eucalyptus miR160* loci and three potential *miR167* loci, all predicted gene products formed typical microRNA stem–loop structures (Fig. S7). The three *EgrmiR160* genes encode a mature RNA identical to that in *Arabidopsis*. The three miR167 genes produce two different mature RNA forms (Table S6) whereas in *Arabidopsis* three different mature miR167 forms were detected. We also identified a potential *TAS3* locus in the *Eucalyptus* genome (Table S6).

We used these newly identified *Eucalyptus* small RNAs as probes to search *in silico* for their target sites in *EgrARF* genes. Ten of the 17 *EgrARF* genes were found to be potential targets of these three small RNAs (Table S7). We identified highly conserved target sites for *EgrmiR160* in *EgrARF10*, *16A*, *16B* and *17*, for *EgrmiR167* in *EgrARF6A* and *B*, and for *EgrTAS3* in *EgrARF2A*, *2B*, *3* and *4* (Table S7). The targeting of three different small RNA to their corresponding target genes was highly conserved between *Arabidopsis* and *Eucalyptus* suggesting common regulation of plant growth and development. For example, *miR160*, a highly conserved miRNA group across the plant kingdom, is known to target *ARF10*, *ARF16* and *ARF17* to regulate various aspects of plant development [30], [52], [53]. In *Arabidopsis*, miR167 regulates lateral root outgrowth [57], adventitious rooting [58], ovule and anther growth, flower maturation [20], [29] and jasmonic acid homeostasis [59] by targeting both *AtARF6* and *AtARF8*. Very recently, it has also been shown that miR167 regulates flower development and female sterility in tomato [60]. Because *Eucalyptus* is a woody perennial plant, one could expect that some small RNAs (for instance miR160 and *Tasi* 3) could be involved in the regulation of wood formation through targeting of ARF genes preferentially expressed in cambial cells or developing xylem.

### Expression of *EgrARFs* in different *Eucalyptus* organs and tissues and in response to environmental cues

**Figure 3:**
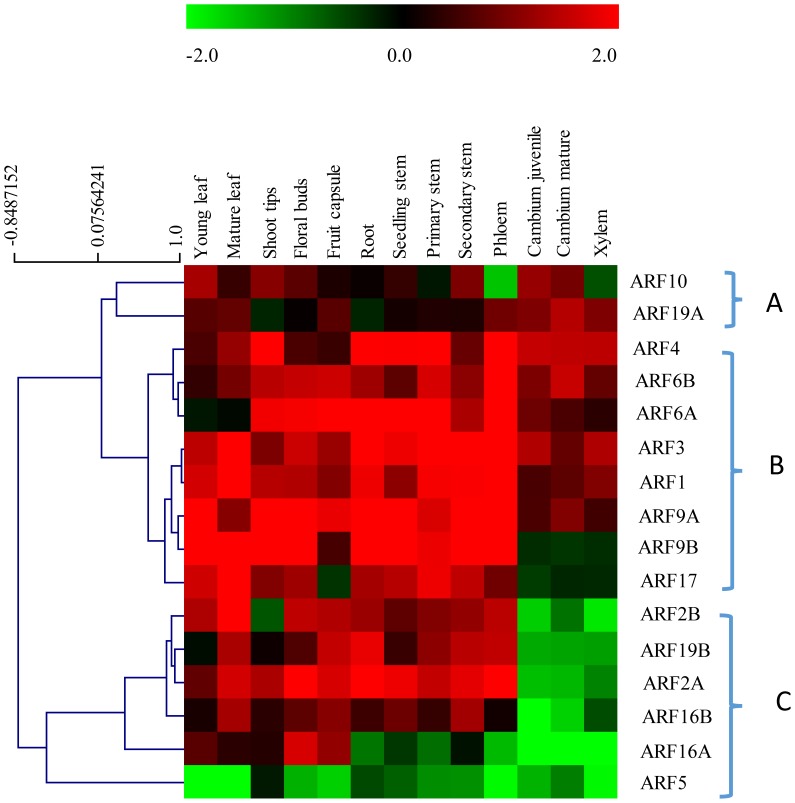
Expression profiles of 16 *EgrARF* genes in various organs and tissues. The heat map was constructed by using the relative expression values determined by qRT-PCR of 16 *EgrARF* genes (indicated on the right) in 13 tissues and organs (indicated at the top) normalized with a control sample (*in vitro* plantlets). In the heat map, red and green indicate relatively high and lower expression (log_2_ratios) than in the control, respectively. Each measurement is the mean of three independent samples. The heat map and the hierarchical clustering were generated by MultiExperiment Viewer (MEV).

To start investigating the functions of the *EgrARF* genes, we assessed their transcript expression levels in various *Eucalyptus* organs and tissues by qRT-PCR, with special attention to vascular tissues (Fig. 3, Fig. S8 and Fig. S9). Transcript accumulation was detected for 16 *EgrARFs* in all 13 organs and tissues tested (Fig. 3), except for *EgrARF24*, which was detected only in shoot tips and young leaves (Fig. S8A). The very restricted expression profile of *EgrARF24* is surprising first because this gene belongs to a woody-preferential clade and second, because its poplar orthologs *PtrARF2.5* and *PtrARF2.6* could be detected in xylem based on microarray expression data [38], *PtrARF2.6* being highly expressed in developing wood (http://popgenie.org/). It should be noted however that this gene is truncated in *E. grandis*, it has lost domain III, whereas *PtrARF2.6* and their grapevine ortholog still have domain III and IV.

Heatmap representation (Fig. 3) indicated that *EgrARF* genes were expressed across various tissues and organs, but different members displayed preference to particular tissues and/or organs and could therefore be clustered into three main expression groups. Group A is the smallest with only two members *EgrARF10* (predicted repressor) and *EgrARF19A* (predicted activator) showing a relatively higher expression in vascular cambium as compared to other tissues and/or organs. *EgrARF10* was expressed at higher level in cambium (both mature and juvenile) than in differentiating xylem and/or phloem (Fig. 3 and Fig. S9). Its ortholog in *Populus*, *PtrARF10.1*, is highly expressed in developing xylem tissues [38], suggesting that *AtARF10* orthologs in trees might be involved in wood cell differentiation having a different/supplementary role as compared to that of the *Arabidopsis* sister pair *AtARF10* – *AtARF16* whose mutants exhibit root cap defects and abnormal root gravitropism [30]. *EgrARF19A* was expressed at similar levels in the three vascular tissues (Fig. 3 and Fig. S9). Group B is the largest with eight genes (*EgrARF4, 6B, 6A, 3, 1, 9A, 9B, 17*) expressed in all tissues including vascular and non-vascular tissues (Fig. 3). The expression of *EgrARF3* and *EgrARF4* is highest in root, stem and phloem and differs from the specific expression of their *Arabidopsis* orthologs *AtARF3* and *AtARF4* associated with developing reproductive and vegetative tissues. This suggests that they might be involved in other processes than the control of the abaxial identity of the gynoecium, and lateral organs shown in *Arabidopsis* [26]. Group C includes six genes (*EgrARF2A*, *2B*, *5*, *16A*, *16B*, *19B*) preferentially expressed in leaves, floral buds and fruits and virtually absent from vascular tissues and particularly from cambium and xylem (Fig. 3 and Fig. S9). As its *Arabidopsis* ortholog, *EgrARF19B* was highly expressed in root [24]. It should be noted that the activator *EgrARF5* is highly expressed in all samples analysed, with the highest expression in *in vitro* plantlets. Because *in vitro* plantlets were used to normalize the expression data in the heatmap, the expression of *EgrARF5* appeared in green in all other samples (Fig. 3). Its expression profile normalized using a different sample is given in Fig. S8.

**Figure 4:**
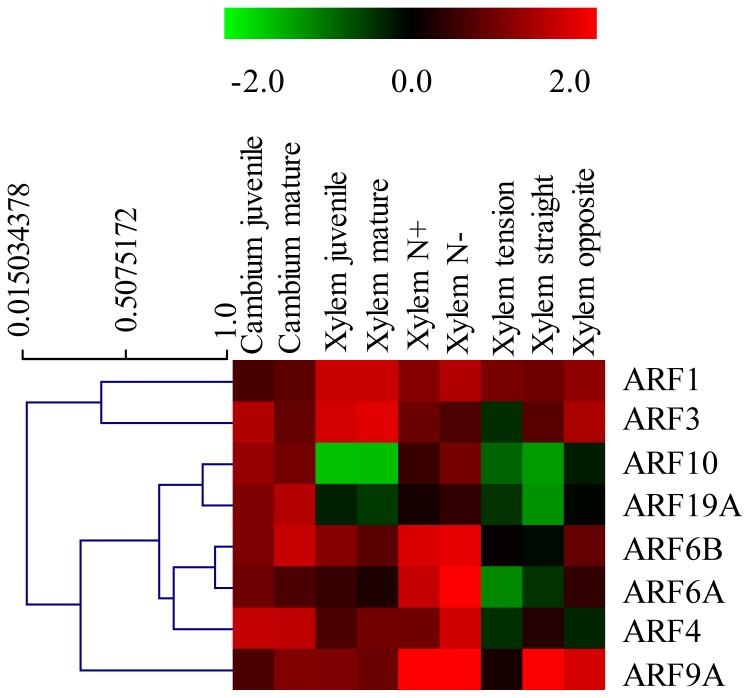
Effect of environmental cues and developmental stages on *EgrARF* expression. The heat map was constructed by using the relative expression values determined by qRT-PCR of *EgrARF* genes (indicated on the right) in various tissues and conditions (indicated at the top) normalized with a control sample (*in vitro* plantlets). In the heat map, red and green indicates relatively higher expression and lower expression (log_2_ratios) than in the control, respectively. The heat map and the hierarchical clustering were generated by MultiExperiment Viewer (MEV).

Thirteen of the sixteen *EgrARF* genes examined (Fig. 3 and Fig. S9) exhibited higher expression in phloem than in xylem and/or cambium, suggesting that in *Eucalyptus* more *EgrARF* genes are involved in phloem than in xylem differentiation and/or function. *EgrARF5* was equally expressed in phloem and xylem. In *Arabidopsis*, *ARF5/MONOPTEROS (MP)* is known to play a critical role in the specification of vascular stem cells [27] but its role in secondary growth driven by vascular cambium activity has not been explored hitherto. *EgrARF10* and *EgrARF19A* were the only two genes more expressed in cambium and/or xylem than in other organs or tissues, supporting their possible involvement during the differentiation of meristematic cambium cells into xylem cells. No obvious difference in transcript levels were observed between juvenile and mature stages neither in cambium nor in differentiating xylem (Fig. 3 and Fig. 4).

We further examined the responsiveness to bending stress of the eight *EgrARF* genes which showed moderate to high expression in vascular tissues (Fig. 4). Half of *EgrARFs* were down-regulated in tension wood as compared to the control upright xylem, including three predicted repressors (*EgrARF3*, *4*, and *9A*) and one predicted activator (*EgrARF6A*). Conversely, in opposite xylem, four genes were up-regulated, including three predicted activators (*EgrARF6A*, *6B*, *19A*) and one repressor (*EgrARF10*). Only one gene (*EgrARF4*) was repressed. In general, *EgrARF* gene expression was repressed in tension xylem and induced in opposite xylem, except in the case of *EgrARF4*, which was down-regulated in both tension and opposite xylem (Fig. 4). These results are consistent with a study performed in *Populus* where seven *ARF* genes were detected in a poplar tension wood EST database, while the majority of genes were down-regulated in tension wood as compared to opposite wood [61].

Recent studies indicated that high nitrogen fertilization affects xylem development and alters fibre structure and composition in *Populus* [62], [63] and induces some overlapping effects with tension wood on xylem cell walls. Interestingly, *EgrARF4* and *EgrARF6A* were down regulated in tension wood, but were down regulated when nitrogen was in excess (Fig. 4).

### Effects of long-term hormone treatments on *EgrARFs* transcript levels

**Figure 5:**
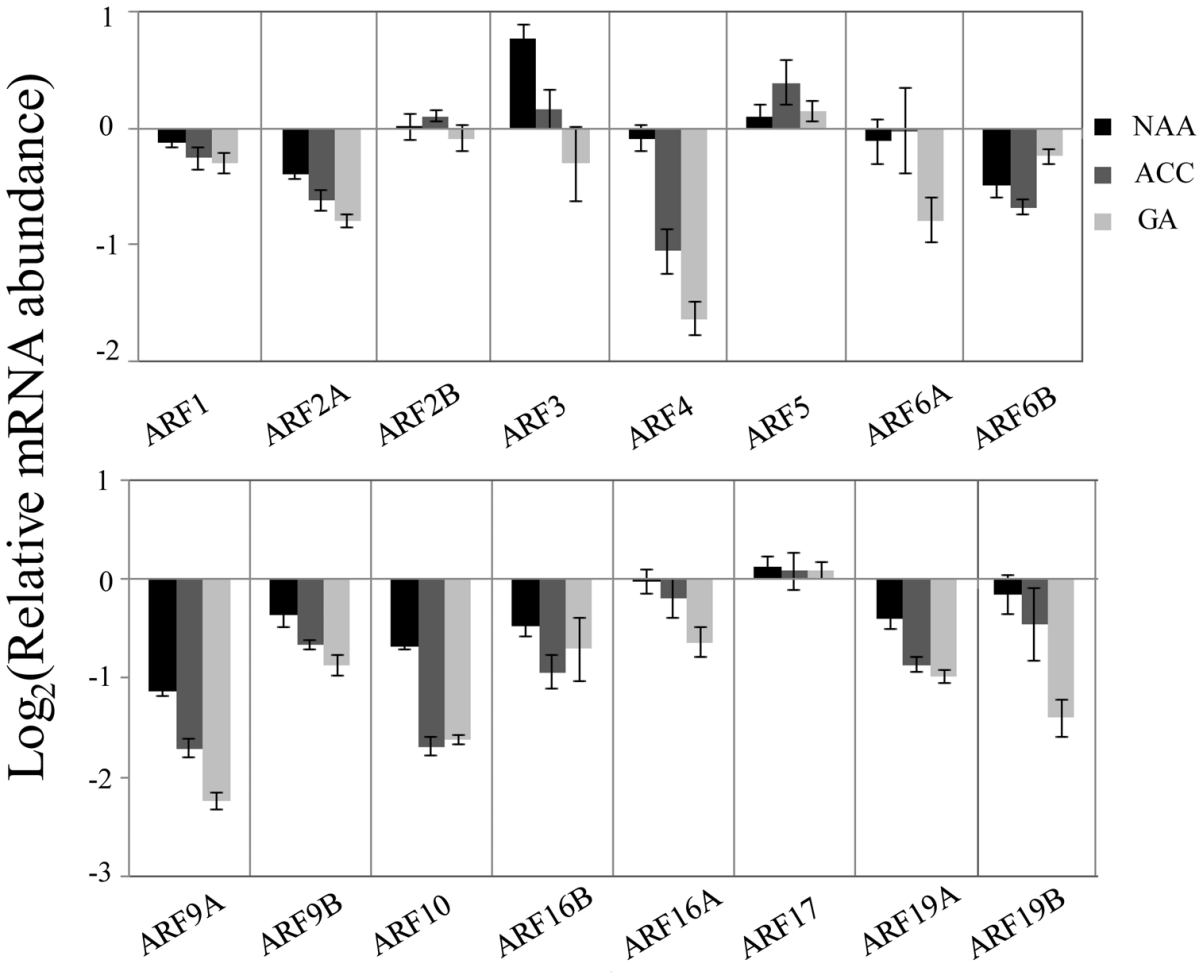
*EgrARF* genes expression levels in young tree stems after long term hormones treatments. Hormone treatments are detailed in the Methods section. NAA, 1-naphthaleneacetic acid, a synthetic auxin usually used in *in vitro* culture. ACC, a precursor of ethylene biosynthesis. GA, gibberellic acid. Relative mRNA abundance was compared to expression in mock-treated young tree stems. Error bars indicated the SE of mean expression values from three independent experiments.

Several hormones are known to regulate cambium activity and xylem formation [[31], [64] and references therein]. For instance, application of exogenous ethylene (ACC) on young poplar trees during 12 days was shown to stimulate cambial activity, while xylem cell size was decreased [65]. We performed similar long-term hormonal treatments (15 days) by growing young *Eucalyptus* trees on medium supplemented with either auxin, gibberellin or ethylene in order to evaluate the consequences on the transcripts levels of the *EgrARF* genes in stems (organs enriched in xylem). The phenotypes of the *Eucalyptus* trees after hormonal treatments were typical of each hormone: gibberellin stimulated plant growth resulting in longer stems, ethylene reduced plant growth and led to epinastic leaves, whereas auxin induced shortened and bolded roots (Fig. S10). All *EgrARF* transcripts except *EgrARF24* were detected in young tree stems and the expression levels of 13 were altered and mainly down-regulated by long-term hormonal treatments (Fig. 5). Although long-term hormonal treatments likely have both direct and indirect effects on *ARFs* expression, it is interesting to note distinct and differential behaviours: Five *ARFs* exhibited a kind of “hormonal preference” response since their transcripts levels were altered in stems treated only by one of the three hormones. For instance, *EgrARF3* was up-regulated only in auxin treated samples; *EgrARF5*, only in ethylene treated samples, whereas *EgrARF6A*, *EgrARF16A* and *EgrARF19B* were altered only in gibberellin treated samples. Most of the other *ARFs* were modulated at different degrees by the direct and/or indirect actions of each of three hormones with the notable exception of *EgrARF4* that was down-regulated in stems treated by ethylene and gibberellic acid but not affected in those treated by auxin.

### Transcriptional activities of EgrARF4, EgrARF 10 and EgrARF19A

**Figure 6:**
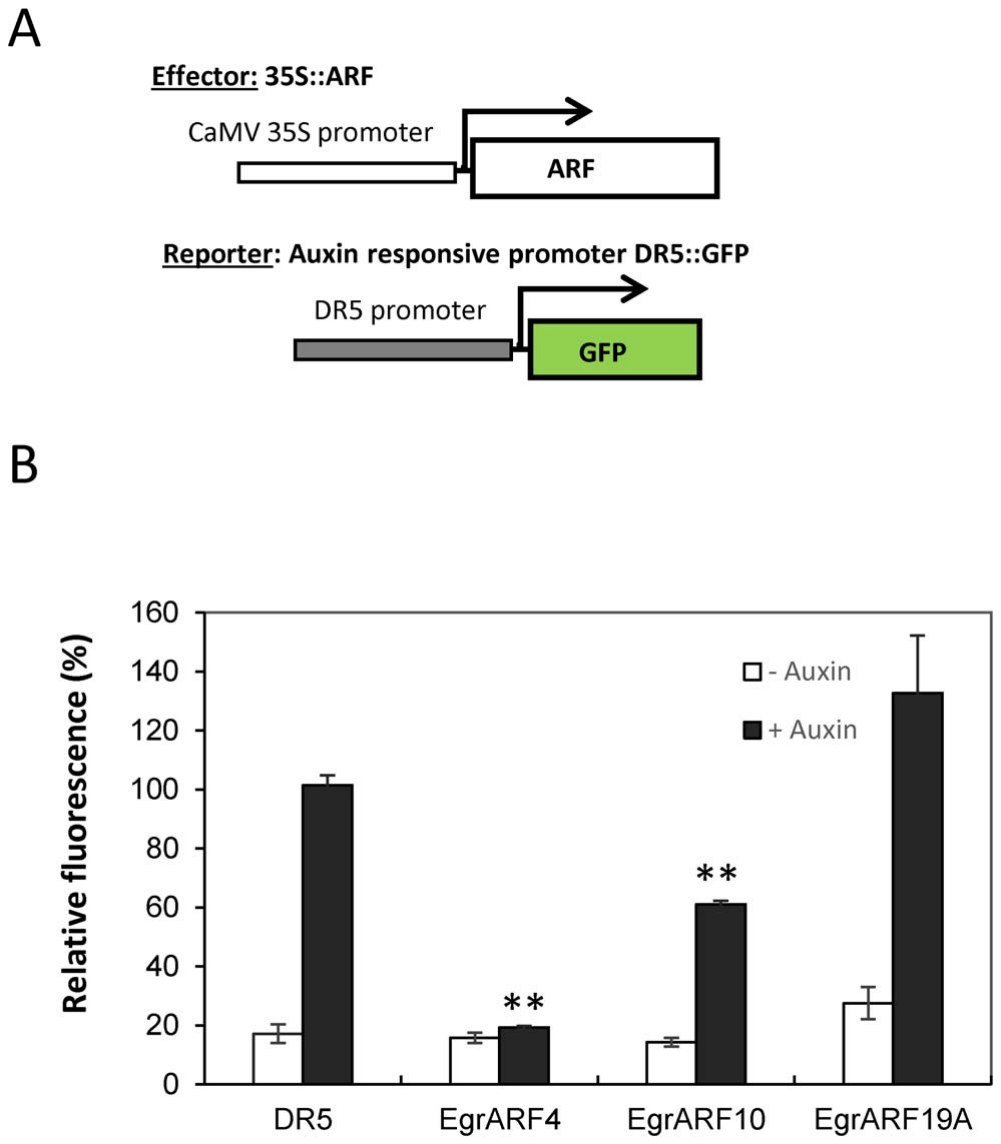
*Egr*ARF transcriptional activities in tobacco protoplasts. (**A**) Schemes of the effector and reporter constructs used to analyse the function of *Egr*ARFs in auxin-responsive gene expression. The effector constructs express the *Egr*ARF of interest driven by the 35S promoter. The reporter construct consists of a reporter gene expressing GFP driven by the auxin-responsive promoter DR5 (DR5::GFP). (**B**) Effector and reporter constructs were co-expressed in tobacco protoplasts in the presence or absence of a synthetic auxin (50 µM 2, 4-D). GFP fluorescence was quantified 16 h after transfection by flow cytometry. A mock effector construct (empty vector) was used as a control. In each experiment, protoplast transformations were performed in independent biological triplicates. Two independent experiments were performed and similar results were obtained; the figure indicates the data from one experiment. Error bars represent SE of mean fluorescence. Significant statistical differences (student T test, P<0.001) to control are marked with **.

We decided to characterize the transcriptional activity of three *ARF* members: *EgARF10* and *19A* which were preferentially expressed in cambium/xylem, and *EgARF4* whose expression was modulated in xylem in response to mechanical stress and to nitrogen fertilization. For this purpose, tobacco protoplasts were co-transfected with an effector construct expressing the full-length coding sequence of the *ARFs* under the *35SCaMV* promoter and a reporter construct carrying the auxin-responsive *DR5* promoter fused to *GFP* coding sequence (Fig. 6A). *DR5* is a synthetic auxin-responsive promoter made of nine inverted repeats of the conserved Auxin-Responsive Element, (TGTCTC box), fused to a *35SCaMV* minimal promoter. This reporter construct has been widely used to assess auxin responsive transcriptional activation or repression *in vivo* and *in planta* [15], [47]. The *DR5*-driven GFP showed low basal activity which was induced up to 4-fold by exogenous auxin treatment (Fig. 6B). Co-transfection with the effector genes *EgrARF4* and *EgrARF10* resulted in a very significant (p<0.001) repression of auxin-induced reporter gene. Expression of 80% and 38%, respectively hereby confirming their predicted repressors roles. On the other hand, the values obtained for *EgrARF19A* suggested that it could be an activator as predicted by its sequence analysis, but this tendency was not strongly supported by the student-T test.

## Conclusions

The *ARF* family in *E. grandis* contains 17 members (5 activators and 12 repressors) and is slightly contracted as compared to most angiosperm *ARF* families studied hitherto. In contrast to these species, it is characterized by the absence of whole genome, segmental and/or tandem duplication events. Indeed, whole genome duplication in *Eucalyptus* occurred 109.9 Mya ago, considerably earlier than those detected in other rosids and 95% of the paralogs were lost [40]. The absence of tandem duplication is remarkable especially because *E. grandis* has the largest number of genes in tandem repeats (34% of the total number of genes) reported among sequenced plant genomes. Indeed, tandem duplication shaped functional diversity in many gene families in *Eucalyptus*. The *ARF* family thus evolved in a very different way. Our data suggests that genomic truncation and alternative splicing were preeminent mechanisms leading to the diversity of domain architecture, shaping and increasing the functional diversity of the *ARF* family in *Eucalyptus*, thereby compensating for the lack of extensive gene duplication found in other species. Comparative phylogenetic studies pointed out the presence of a new clade, maintained preferentially in woody and perennial plants. Finally, large scale expression profiling allowed identifying candidates potentially involved in the auxin-regulated transcriptional programs underlying wood formation.

## Supporting Information

Figure S1 **Procedure used for identifying** ***ARF*** **genes in** ***Eucalyptus grandis*** **.** *Arabidopsis* ARF protein sequences were used to search their orthologs in the predicted *Eucalyptus* proteome by using in BLASTP. Sixty-four *Eucalyptus* proteins identified in this initial search were further examined by manual curation using protein motif scanning and the FgeneSH program to complete partial sequences. Redundant and invalid genes were eliminated based on gene structure, integrity of conserved motifs and EST support. Manual curation resulted in 17 complete Eucalyptus ARF protein sequences. These 17 protein sequences were used in two subsequent additional searches: first, a BLASTP search against the *Eucalyptus* proteome to identify exhaustively all divergent *Eucalyptus ARF* gene family members and, second, tBLASTn searches against the *Eucalyptus* genome for any possible unpredicted genes. To confirm our findings, we used poplar ARF proteins and repeated the complete search procedure described above and obtained identical results.
(TIFF)

Figure S2 **Locations of the 17** ***EgrARF*** **genes on the 11** ***Eucalyptus grandis*** **chromosomes.**
(TIFF)

Figure S3 **Multiple sequence alignment of predicted amino acid sequences of** ***EgrARF*** **and** ***AtARF*** **proteins.** The multiple sequence alignment was obtained with the MUSCLE software [66]. The highly conserved domains and nuclear localization signals (NLSs) proteins were noted on the bottom of the alignment with different colours.
(PDF)

Figure S4 **Comparative analysis of predicted** ***ARF*** **alternative variants between** ***Eucalyptus grandis*** **and** ***Arabidopsis thaliana*** **.** The alternative spliced protein sequences were extracted from Phytozome except for *AtARF4* (obtained from Finet *et al.* (2013), the motif structures were predicted by Pfam (http://pfam.xfam.org/).
(PDF)

Figure S5 **Structure of the** ***ARF*** **alternative transcripts in** ***E. globulus*** **.** The *E. globulus* alternative transcripts were obtained from a compendium of RNASeq data. The material and methods are described in Table S4. The illumina reads sequences are provided in File S1 in the FastQ format.
(PDF)

Figure S6 **Comparative Phylogenetic relationships between ARF proteins from poplar,** ***Eucalyptus*** **, grapevine,** ***Arabidopsis*** **, tomato and rice.** Full-length protein sequences were aligned using the Clustal_X program. The phylogenetic tree was constructed by using the MEGA5 program and the neighbour-joining method with predicted full-length ARF proteins. Bootstrap supports are indicated at each node.
(PDF)

Figure S7 **Predicted stem-loop structures of three** ***EgrmiR160*** **and three** ***EgrmiR167*** **.** The part of the stem-loop from which the mature microRNA derives is highlighted in yellow.
(TIFF)

Figure S8 **Expression profiles of** ***EgrARF5*** **and** ***EgrARF24*** **in various organs and tissues.** Relative mRNA abundance of *EgrARF5* and *EgrARF24* was compared to expression in the control sample of mature leaves and *in vitro* plantlets, respectively. Error bars indicate the SE of mean expression values from three independent experiments.
(TIFF)

Figure S9 **Expression profiles of** ***EgrARF*** **genes in tissues involved in secondary growth.** Relative mRNA abundance was compared to expression in the control sample (*in vitro* plantlets).
(TIFF)

Figure S10 **Young** ***Eucalyptus grandis*** **trees phenotypes in response to various long-term hormonal treatments.** 10 µM NAA, or 20 µM gibberellic acid or 100 µM ACC were added to the medium of 65-d-old young tree, and phenotypes were observed 14 days later.
(TIFF)

Table S1 **Primers for** ***EgrARF*** **genes and reference genes used in qRT-PCR experiments.**
(PDF)

Table S2 **Protein identity matrix between** ***Egr*** **ARF and** ***At*** **ARF.**
(PDF)

Table S3 **Protein identity matrix among** ***Egr*** **ARF.**
(PDF)

Table S4 **Comparison of the number of alternative transcripts predicted in phytozome** ***for E. grandis*** **to those found in a large compendium of transcriptomic data from in** ***E. globulus*** **.**
(PDF)

Table S5 **Comparison of the number of** ***EgrARF24*** **putative orthologs in other species.**
(PDF)

Table S6 **Potential small RNAs targeting** ***EgrARF*** **genes.**
(PDF)

Table S7 **Small RNAs target site prediction in** ***EgrARF*** **genes.**
(PDF)

File S1 **Sequences of the Illumina reads from RNA Seq used to predict the** ***E. globulus*** **alternative transcripts.** The origin of the material and the procedure are described in Table S4.
(ZIP)
