# Supplementary figures and images for: Genome-Wide Characterization and Expression Profiling of the AUXIN RESPONSE FACTOR (ARF) Gene Family in Eucalyptus grandis
Source: PLoS One. 2014 Sep 30;9(9):e108906. doi: 10.1371/journal.pone.0108906 (PMC4182523; doi:10.1371/journal.pone.0108906)

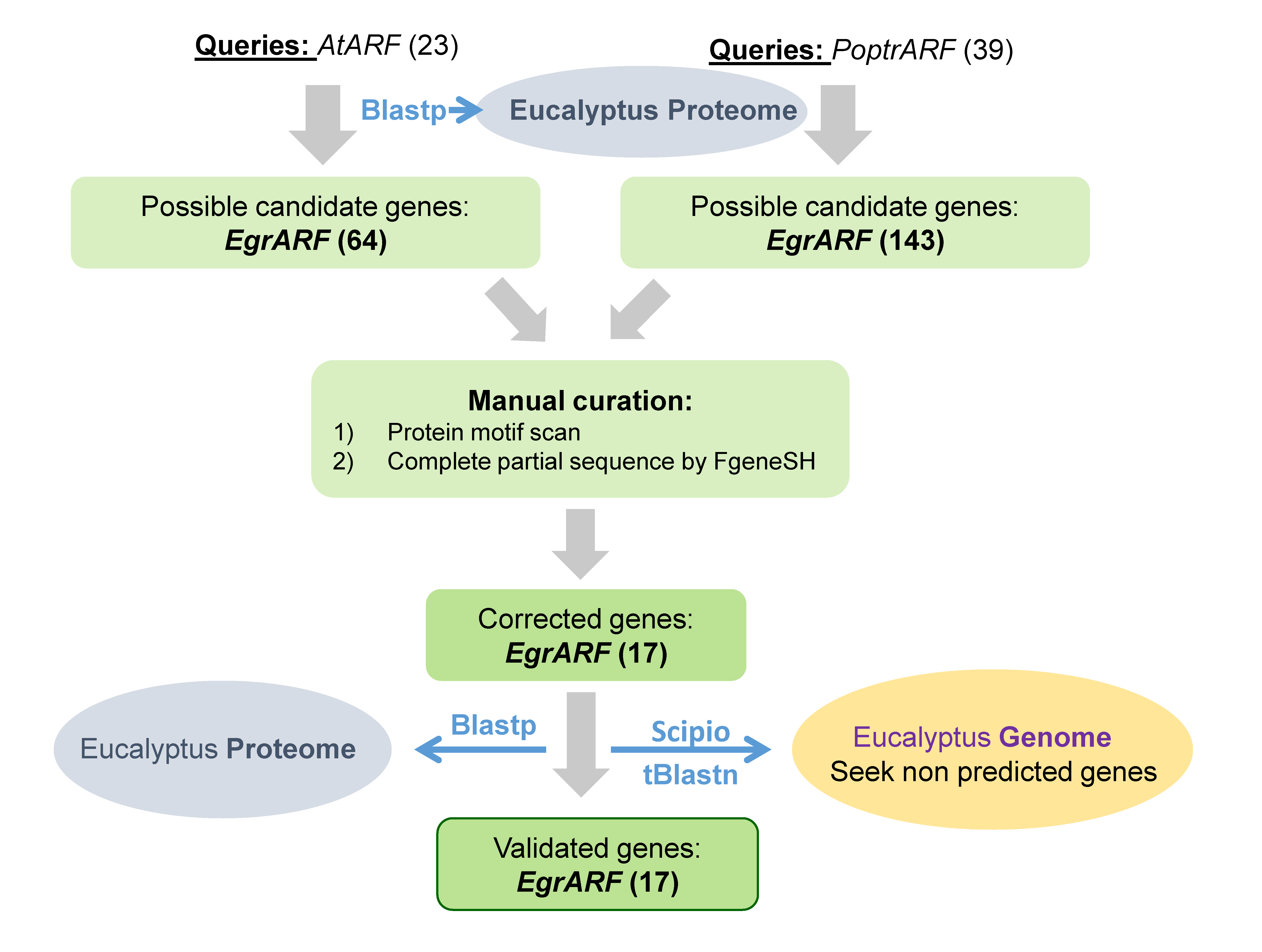

Supplement: Figure S1 — Procedure used for identifying ARF genes in Eucalyptus grandis . Arabidopsis ARF protein sequences were used to search their orthologs in the predicted Eucalyptus proteome by using in BLASTP. Sixty-four Eucalyptus proteins identified in this initial search were further examined by manual curation using protein motif scanning and the FgeneSH program to complete partial sequences. Redundant and invalid genes were eliminated based on gene structure, integrity of conserved motifs and EST support. Manual curation resulted in 17 complete Eucalyptus ARF protein sequences. These 17 protein sequences were used in two subsequent additional searches: first, a BLASTP search against the Eucalyptus proteome to identify exhaustively all divergent Eucalyptus ARF gene family members and, second, tBLASTn searches against the Eucalyptus genome for any possible unpredicted genes. To confirm our findings, we used poplar ARF proteins and repeated the complete search procedure described above and obtained identical results. (TIFF) [file pone.0108906.s001.tiff]

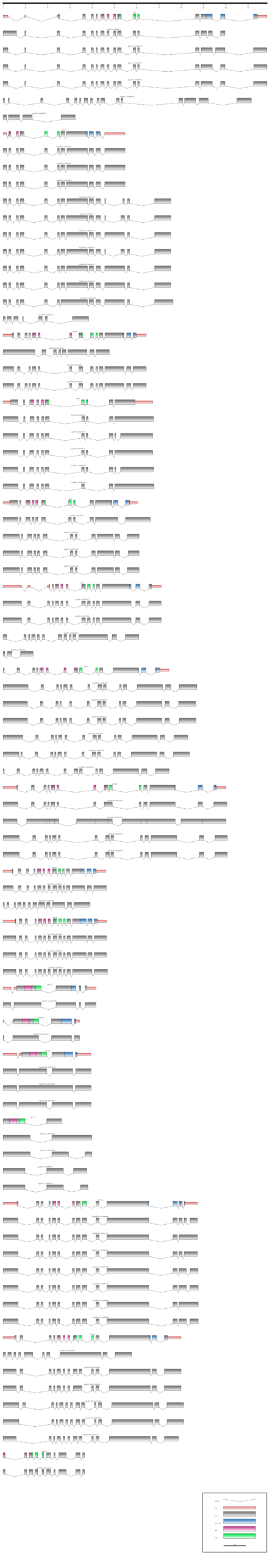

Supplement: Figure S5 — Structure of the ARF alternative transcripts in E. globulus . The E. globulus alternative transcripts were obtained from a compendium of RNASeq data. The material and methods are described in Table S4. The illumina reads sequences are provided in File S1 in the FastQ format. (PDF) [file pone.0108906.s005.pdf]

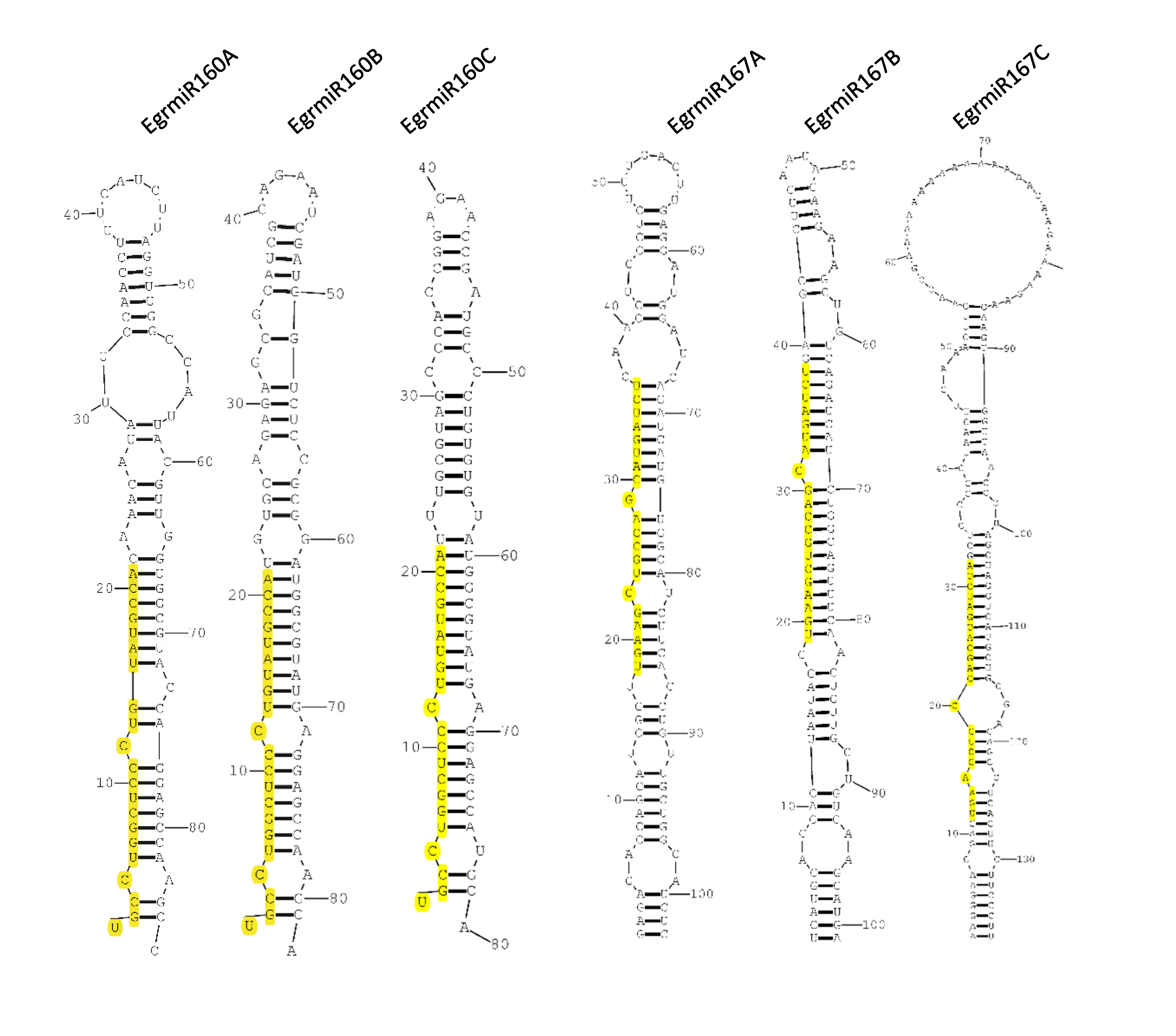

Supplement: Figure S7 — Predicted stem-loop structures of three EgrmiR160 and three EgrmiR167 . The part of the stem-loop from which the mature microRNA derives is highlighted in yellow. (TIFF) [file pone.0108906.s007.tiff]

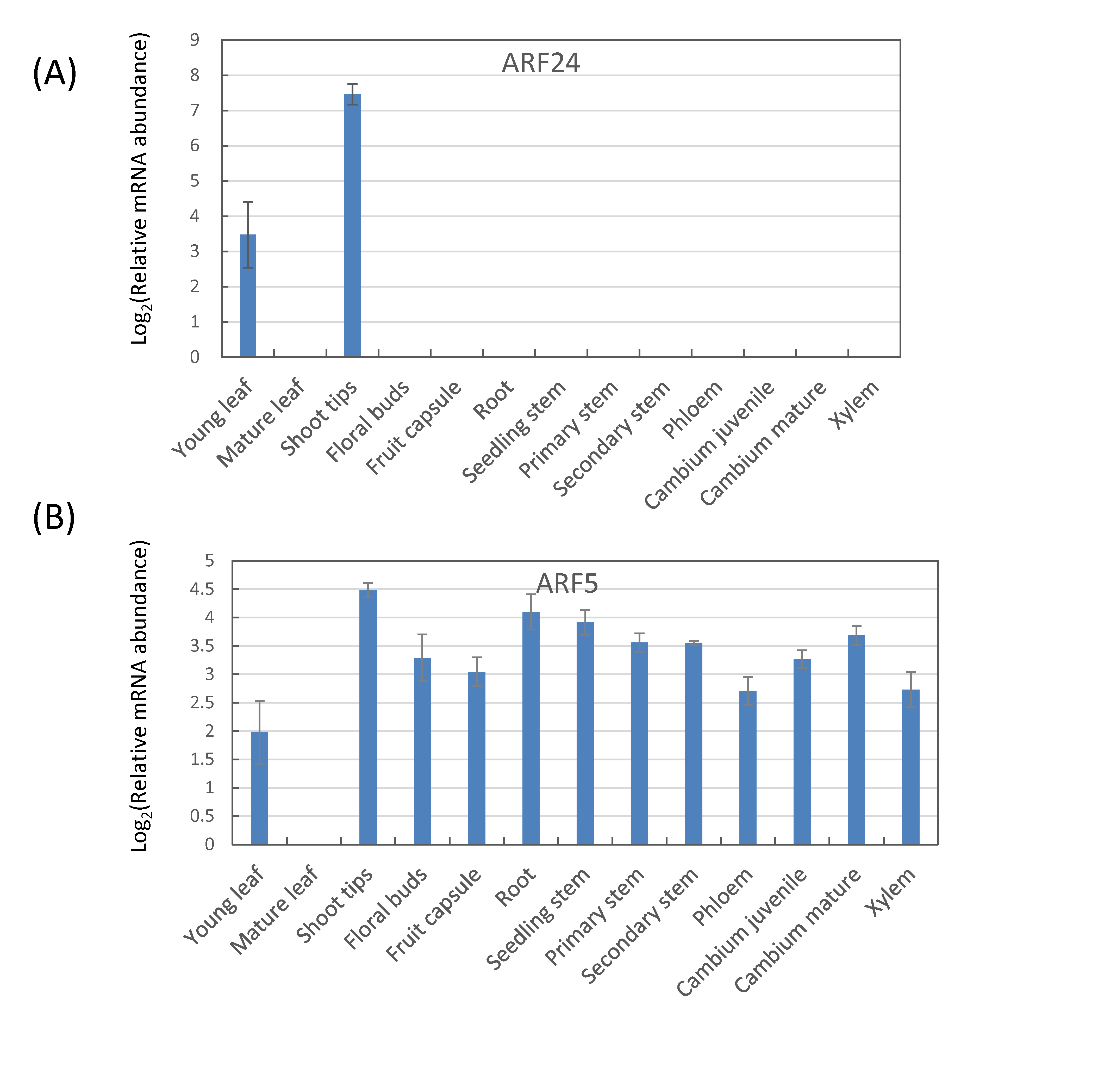

Supplement: Figure S8 — Expression profiles of EgrARF5 and EgrARF24 in various organs and tissues. Relative mRNA abundance of EgrARF5 and EgrARF24 was compared to expression in the control sample of mature leaves and in vitro plantlets, respectively. Error bars indicate the SE of mean expression values from three independent experiments. (TIFF) [file pone.0108906.s008.tiff]

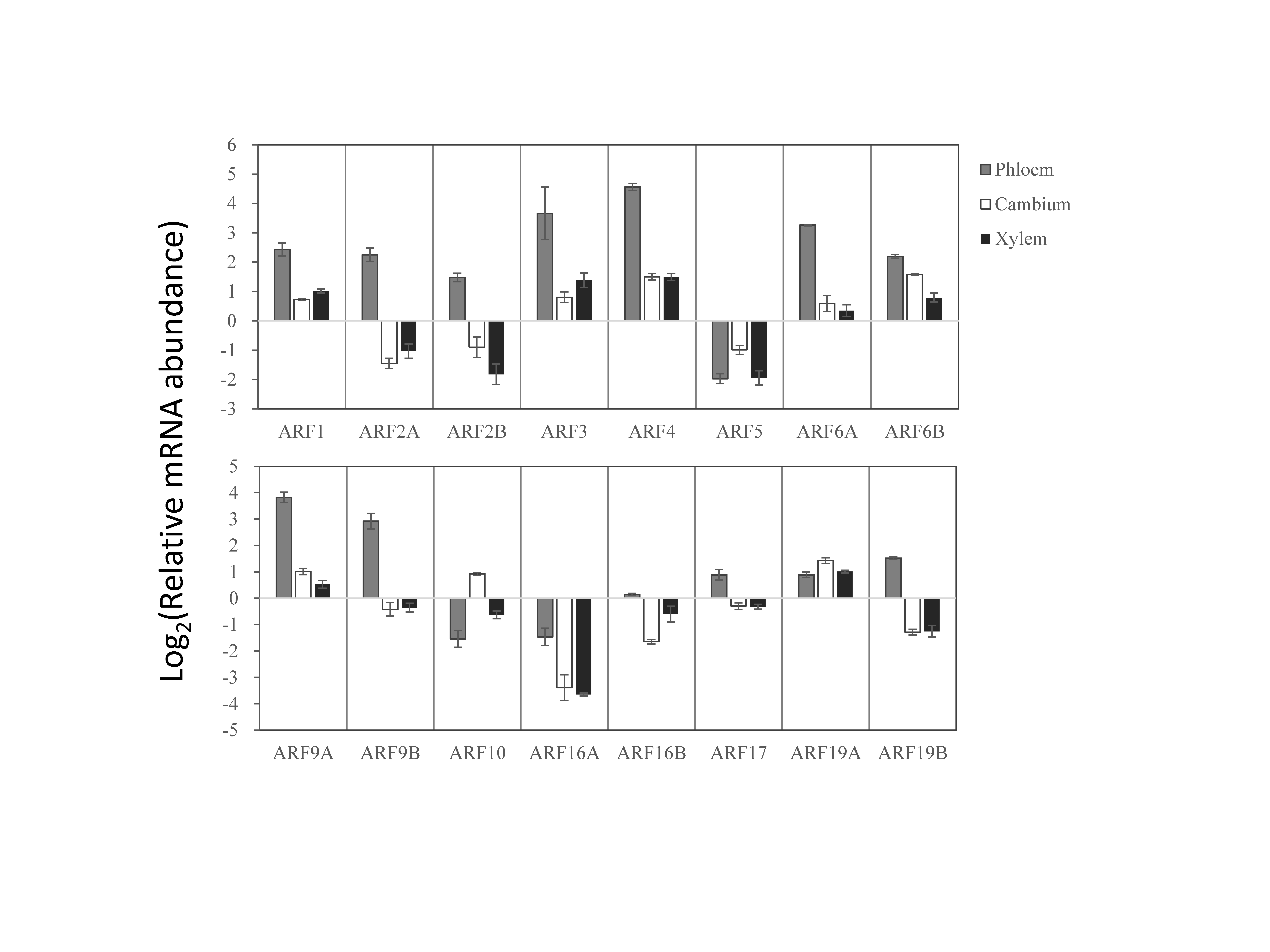

Supplement: Figure S9 — Expression profiles of EgrARF genes in tissues involved in secondary growth. Relative mRNA abundance was compared to expression in the control sample (in vitro plantlets). (TIFF) [file pone.0108906.s009.tiff]

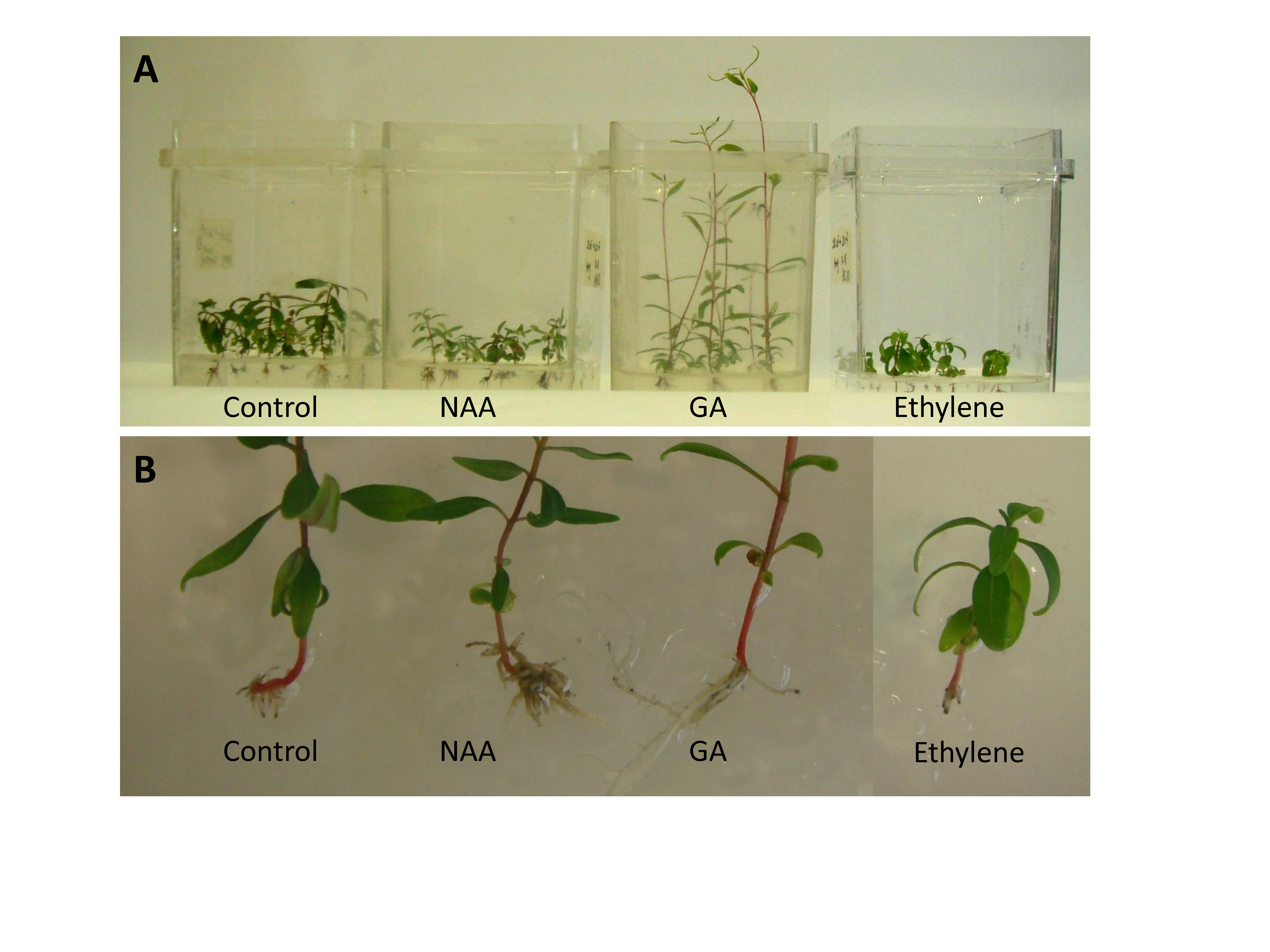

Supplement: Figure S10 — Young Eucalyptus grandis trees phenotypes in response to various long-term hormonal treatments. 10 µM NAA, or 20 µM gibberellic acid or 100 µM ACC were added to the medium of 65-d-old young tree, and phenotypes were observed 14 days later. (TIFF) [file pone.0108906.s010.tiff]
