# Supplementary material for: Genome-Wide Characterization and Expression Profiling of the AUXIN RESPONSE FACTOR (ARF) Gene Family in Eucalyptus grandis
Source: PLoS One. 2014 Sep 30;9(9):e108906. doi: 10.1371/journal.pone.0108906 (PMC4182523; doi:10.1371/journal.pone.0108906)
Supplement: Figure S3 — Multiple sequence alignment of predicted amino acid sequences of EgrARF and AtARF proteins. The multiple sequence alignment was obtained with the MUSCLE software [66]. The highly conserved domains and nuclear localization signals (NLSs) proteins were noted on the bottom of the alignment with different colours. (PDF) [file pone.0108906.s003.pdf]

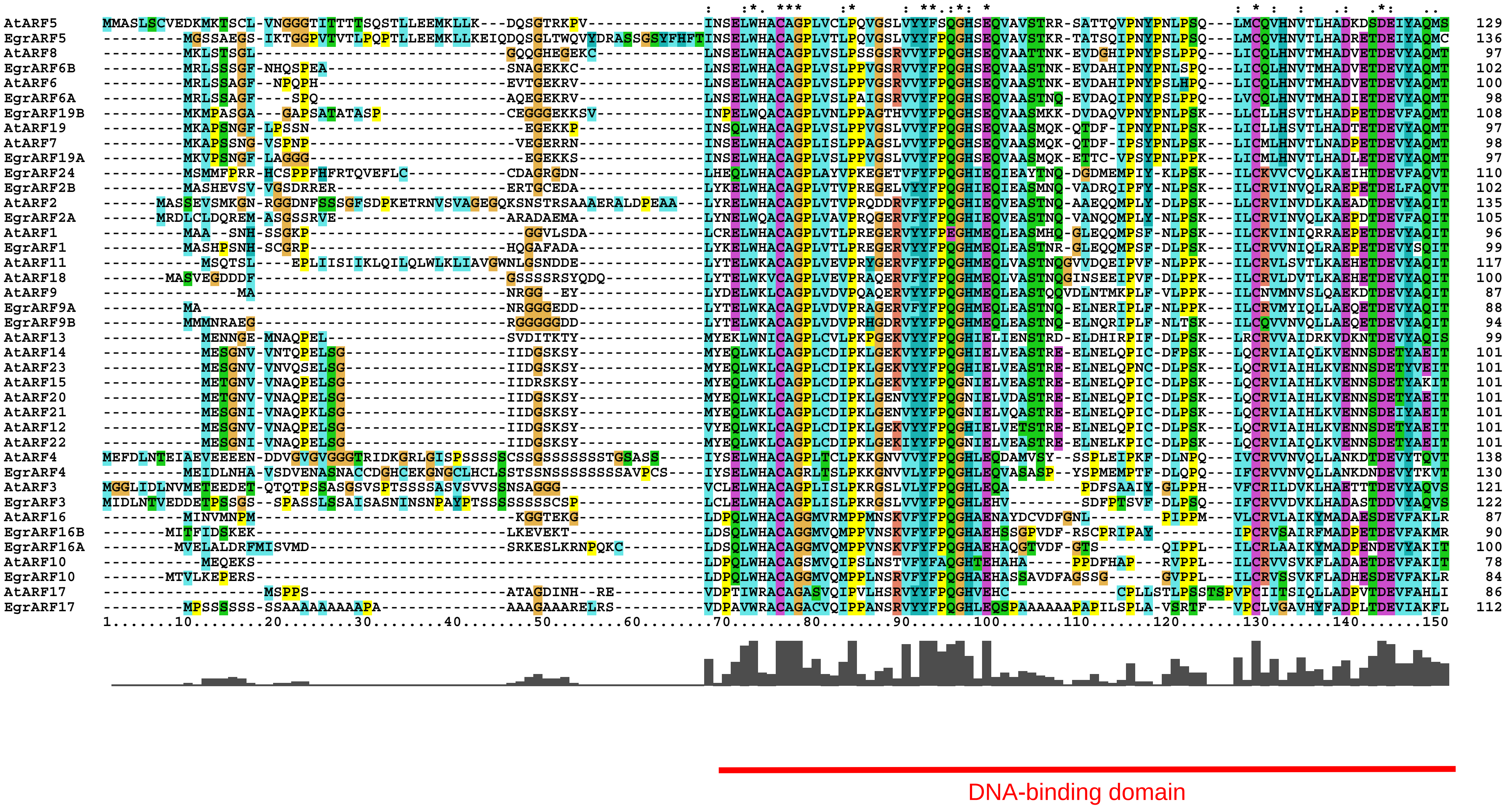
AtARF5 129
EgrARF5 136
AtARF8 97
EgrARF6B 102
AtARF6 100
EgrARF6A 98
EgrARF19B 108
AtARF19 97
AtARF7 98
EgrARF19A 97
EgrARF24 110
EgrARF2B 102
AtARF2 135
EgrARF2A 105
AtARF1 96
EgrARF1 99
AtARF11 117
AtARF18 100
AtARF9 87
EgrARF9A 88
EgrARF9B 94
AtARF13 99
AtARF14 101
AtARF23 101
AtARF15 101
AtARF20 101
AtARF21 101
AtARF12 101
AtARF22 101
AtARF4 138
EgrARF4 130
AtARF3 121
EgrARF3 122
AtARF16 87
EgrARF16B 90
EgrARF16A 100
AtARF10 78
EgrARF10 84
AtARF17 86
EgrARF17 112
DNA-binding domain

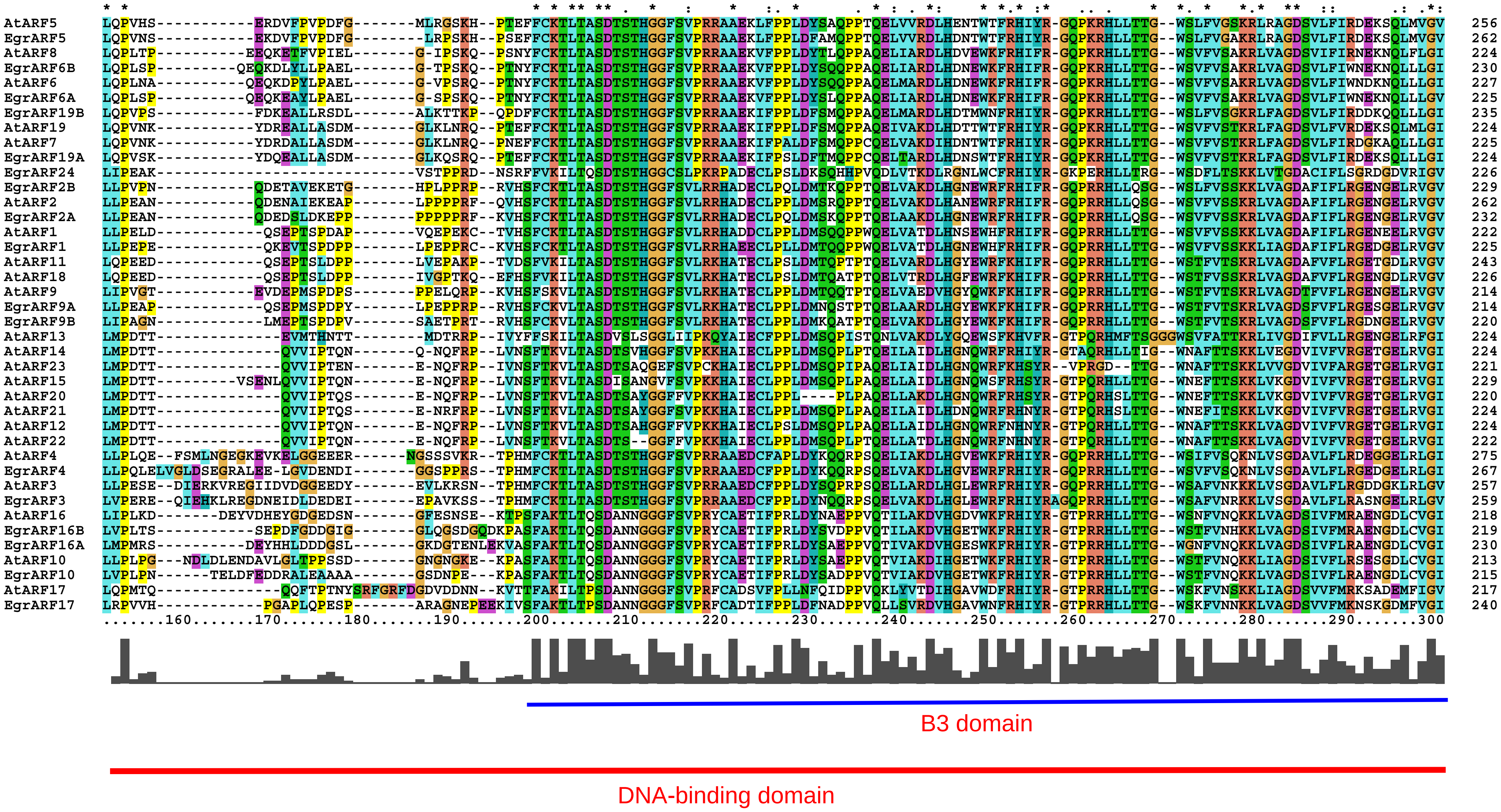
AtARF5
EgrARF5
AtARF8
EgrARF6B
AtARF6
EgrARF6A
EgrARF19B
AtARF19
AtARF7
EgrARF19A
EgrARF24
EgrARF2B
AtARF2
EgrARF2A
AtARF1
EgrARF1
AtARF11
AtARF18
AtARF9
EgrARF9A
EgrARF9B
AtARF13
AtARF14
AtARF23
AtARF15
AtARF20
AtARF21
AtARF12
AtARF22
AtARF4
EgrARF4
AtARF3
EgrARF3
AtARF16
EgrARF16B
EgrARF16A
AtARF10
EgrARF10
AtARF17
EgrARF17
B3 domain
DNA-binding domain

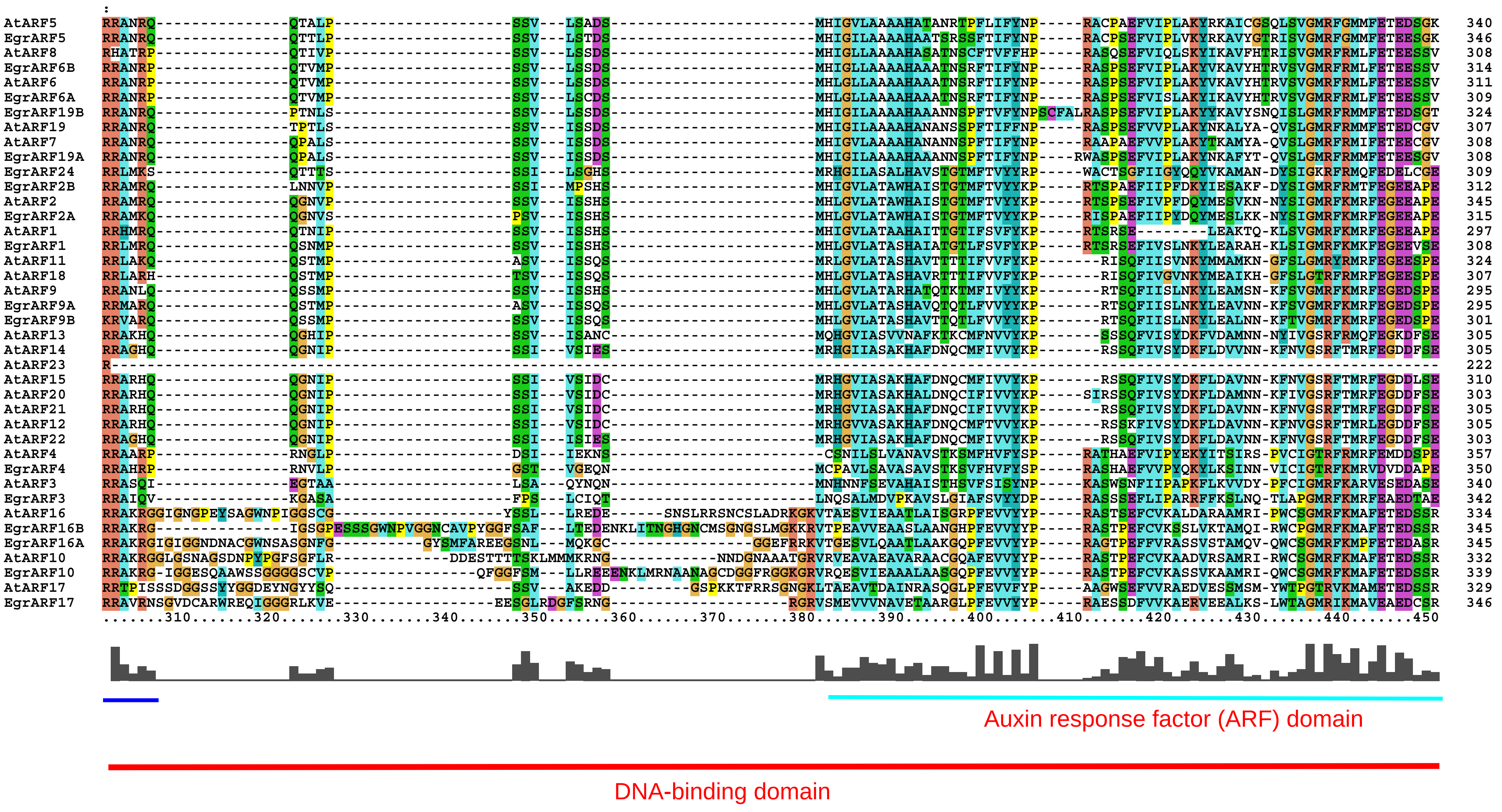
AtARF5
EgrARF5
AtARF8
EgrARF6B
AtARF6
EgrARF6A
EgrARF19B
AtARF19
AtARF7
EgrARF19A
EgrARF24
EgrARF2B
AtARF2
EgrARF2A
AtARF1
EgrARF1
AtARF11
AtARF18
AtARF9
EgrARF9A
EgrARF9B
AtARF13
AtARF14
AtARF23
AtARF15
AtARF20
AtARF21
AtARF12
AtARF22
AtARF4
EgrARF4
AtARF3
EgrARF3
AtARF16
EgrARF16B
EgrARF16A
AtARF10
EgrARF10
AtARF17
EgrARF17
Auxin response factor (ARF) domain
DNA-binding domain

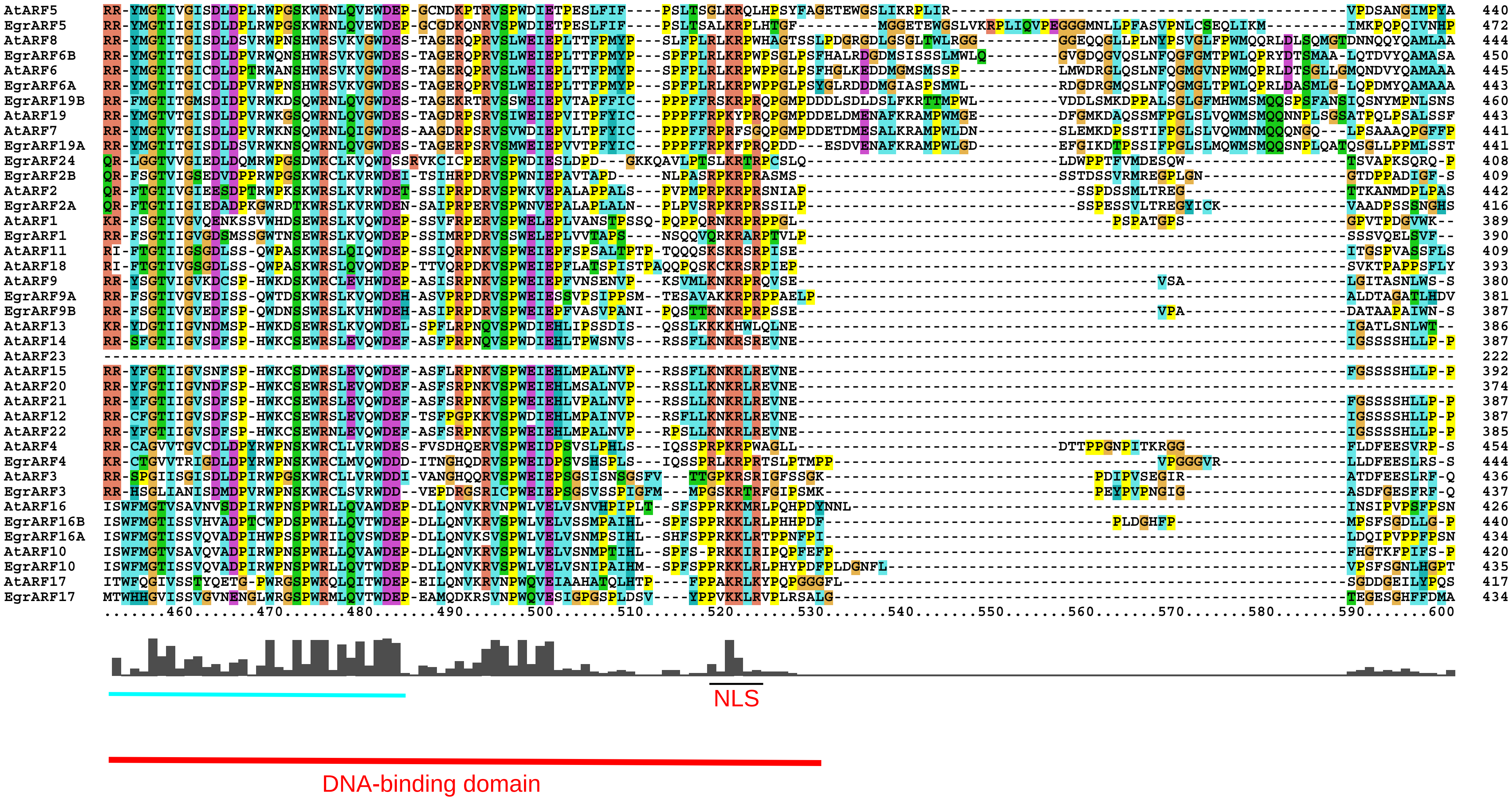

AtARF5 440
EgrARF5 472
AtARF8 444
EgrARF6B 450
AtARF6 445
EgrARF6A 443
EgrARF19B 460
AtARF19 443
AtARF7 441
EgrARF19A 441
EgrARF24 408
EgrARF2B 409
AtARF2 442
EgrARF2A 416
AtARF1 389
EgrARF1 390
AtARF11 409
AtARF18 393
AtARF9 380
EgrARF9A 381
EgrARF9B 387
AtARF13 386
AtARF14 387
AtARF23 222
AtARF15 392
AtARF20 374
AtARF21 387
AtARF12 387
AtARF22 385
AtARF4 454
EgrARF4 444
AtARF3 436
EgrARF3 437
AtARF16 426
EgrARF16B 440
EgrARF16A 434
AtARF10 420
EgrARF10 435
AtARF17 417
EgrARF17 434
460 470 480 490 500 510 520 530 540 550 560 570 580 590 600
NLS
DNA-binding domain

```
AtARF5     SFPS---------MASEQLMKMMMRPHNNQNVP----------------------------------------SFMSEMQQNIVMGNGGLLGDMK-------------------------MQQPLMMNQKSEMVQPQNK-------LTVN   509
EgrARF5    SLP----------QDNNPKFIPGHEVNTMQGTV----------------------------------------NQKPQAFNTESAIPPKLIYPHLCPDQSSAMGLSSEVHVTNKVPPPYKTENQAPVETKDEKLNVDSH-------LKKD   565
AtARF8     GLQNI--------GGGDPLRQQFVQLQEPHHQY----------------------------------------LQQSASHNSDLM-----------------------------------LQQQQQQQASRHLMHAQTQ-------IMSE   504
EgrARF6B   ALQDM--------RAVDPSKCASQSLLPLQQSQ---------------------------------------NVPMGQASIIQRQMLQQSQ-----------------------------SQNSLLQGFQENQATAQGQ-------VLQH   517
AtARF6     ALQDM--------RGIDPAKAAASLLQFQNSPG----------------------------------------FSMQSPSLVQPQMLQQQLSQQQQ------------------------QLSQQQQQQQLSQQQQQQQ-------LSQQ   516
EgrARF6A   ALQEM--------RAVDHSKLATASLMPVQHVQ-----------------------------NITSASASLMPSQMLISTQPQQSLLQGSRESPHHSVSQDQVQSHLLQRQLQHQNSFTNQQQPQQEQLRQQLVNQEQI------PSASS   550
EgrARF19B  VLQNLSGTDPSRQLGFSAPQMAQLNNLQFNTQRPPQQAQQLDQLTKLPSTLNPLSNIVQPQQQLTDISQQSRQSLINHTLPSTQVQAQVLQSQSLIQSSTLLPQQSLNQNQPFHRSLPQNLHQPQQQQIMGQNQQQSAL-------NSQM   603
AtARF19    NLPNN--------FASNDPSKLLNFQSPNLSSA---------------------------------------NSQFNKPNTVNHISQQMQAQPAMVKSQQQQQQQQQQHQHQQQQLQQQQQLQMSQQQVQQQGIYNNGTIAVANQVSCQS   546
AtARF7     SMLSPTAALHNNLGGTDDPSKLLSFQTPHGGISSSNLQFNKQNQQAPMSQLPQPPTTLSQQQQLQQLLHSSLNHQQQQSQSQQQQQQQLLQQQQQLQSQQHSNNNQSQSQQQQQLLQQQQQQQLQQQHQQPLQQQTQQ-----QQLRTQ   586
EgrARF19A  GLHNN--------LGIDDPSKLLSFQAPTQGLQ---------FNKTNPQNQVSQLLQPSMAWSQQHQLQQLLQNPLGHQQQQQQQQLQRQQQQQLQSSQQQTPQQLPQSQPPQQQPLQPQQFQQQSPHQPSQQTSQQQQ-------VVQT   567
EgrARF24   GVF----------QGDG----------GKRESR----------------------------------------GPGDH--------------------------------------------WP--PASIAPGCNPVKS-------LPAQ   445
EgrARF2B   KAL----------QGQEFST-----LKGSLTLN----------------------------------------NDMENAEMP--------------------------------------DAWYEFPGDNRKSMHFGQR-------KCRP   459
AtARF2     GLSRVL-------QGQEY-----STLRTKHTES----------------------------------------VECDAPENS--------------------------------------VVWQSSADDDKVDVVSGSR-------RYGS   495
EgrARF2A   RIL----------QGPESSTLRGIATDNDFDVV--------------------------------------EKSVMFTPSMEEEKIDMLSAS----------------------------KRHGLDSWMTSGRRGPTCADLLSGFGGNTD   490
AtARF1     -------------------------------------------------------------------------SPADTPSS--------------------------------------------VPLFSPPAKAATFG-------HGGN   415
EgrARF1    -------------GGPKAPEYSSDFLHGDSQRG---------------------------------------------------------------------------------------RDVYLSPKFSPSARSKSLN-------YNGN   433
AtARF11    SFS----------QSHES-----------------------------------------------------------------------------------------------------------NPSVKLLFQDPATE-------RNSN   435
AtARF18    SLP-----------------------------------------------------------------------------------------------------------------------------QSQDSINASLK-------LFQD   411
AtARF9     VLT----------QPHEFAQSCITSQWSSPQQC--------------------------------------------------------------------------------------------HRDANEDAKKSDWL-------NNSY   421
EgrARF9A   GLM----------QSCDS-TQPTVFIEGKRSDN------------------------------------------------------------------------------------------HGIWHHKQTSVNTGSN-------SISW   423
EgrARF9B   GYT----------QTHDL---------KQLRVT----------------------------------------DEGKSSD-H--------------------------------------FMWNYKMADVNSSSSSITR-------VQTE   432
AtARF13    --------------CQEI---------GQRSMN----------------------------------------SPISVPEFS-------------------------------------------YPNAIEDSKFLSGL-------LLNH   423
AtARF14    TLT----------QGQEI---------GQQSMA----------------------------------------TPMNISLR--------------------------------------------YRDITEDAMTPSRL-------LMSY   427
AtARF23    ------------------------------------------------------------------------------------------------------------------------------------------------------   222
AtARF15    ILT----------QGQEI---------GQLSVA----------------------------------------SPMNISLL--------------------------------------------YRETTEDAMNPSRL-------LMSY   432
AtARF20    -------------FGQEI---------GQLSVA----------------------------------------SPMNTSLR--------------------------------------------YRDTTEDAMNPSRL-------LMSY   411
AtARF21    ILT----------QGQEI---------GQLSVA----------------------------------------SPMNISLR--------------------------------------------YRDTTEAAMNPSRL-------LMSY   427
AtARF12    ILT----------QGQEN---------EQLSVA----------------------------------------SPMNISLR--------------------------------------------YRDATEDAMNPSKL-------LMSY   427
AtARF22    ILT----------QGQEI---------GQLSVA----------------------------------------SPMNISLT--------------------------------------------YRDTTEDVMNPSRL-------LMSY   425
AtARF4     KVL----------QGQEN--------IGSASPS----------------------------------------QGFDVMNRRILDFAMQSHANPVLVSSR------------------VKDRFGEFVDATGVNPACSGV-------MDLD   521
EgrARF4    KVL----------QGQEK-LHLVSPVYGRDTLN----------------------------------------CQVDFEQSPAHQGLASVVVE---------------------------KANNINEYMRANAPSYAGF-------VESD   509
AtARF3     RVL----------QGQEIFP-------GFINTC-----------------------------------------------------------------------------------------SDGGAGARRGRFKGTEF-------GDSY   473
EgrARF3    KVL----------QGQE----------TFANPP------------------------------------------------------------------------------------------YTRPWFTSGAQGNGAF-------GVRD   470
AtARF16    PLI----------RSSPLSSVLDNVPVGLQGAR---------------------------------------------------------------------------------------HNAHQYYGLSSSDLHHYYL-------NRPP   472
EgrARF16B  SSP----------FGCLPNSHSAAGMQGARHAY----------------------------------------YSQSLPDL--------------------------------------------HLSKQPSSLFPAGF-------PLLD   489
EgrARF16A  PLG----------PNSPLCFIPDNIPAGIQGAR-------------------------------------------------------------------------------------HAQFLRSSSDLRLNQLHSGLF------PVGSH   483
AtARF10    GFANN--------GGGESMCYLSNDNNNAPAGI------------------------------------------------------------------------------------------QGARQAQQLFGSPSPS-------LLSD   465
EgrARF10   GLLCC--------LSDNAPAGIQGARQAQFGIP---------------------------------------------------------------------------------------LTDHHLNNRLRLGLLSSNL-------HRFD   483
AtARF17    GLS----------SAAAPDPSPSMFSYSTFPAG-----------------------------------------------------------------------------------------MQGARQYDFGSFNPTGF-------IGGN   461
EgrARF17   GFT--------------------NPMVPYSSSF--------------------------------------------------------------------------------------------FNYNTPIGMQGARQ-----------   461
           .......610.......620.......630.......640.......650.......660.......670.......680.......690.......700.......710.......720.......730.......740.......750
```

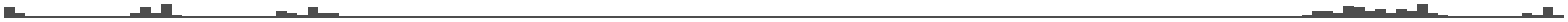

```
AtARF5      PSASNTSGQEQNLSQSMSAP-----------------------------------------------------AKPENSTLSGCSSGRVQHGLEQSMEQ--------------------------------------------------- 555
EgrARF5     VLNQVPSTSTGELPEENIAS------------------------------------------------GPSSQQNHLNHPIILNQNQGQIQSQACSWPP--------------------------------------------------- 616
AtARF8      NLPQQNMRQEVSNQPAGQQQ---------------------------------------------------QLQQPDQNAYLNAFKMQNGHLQQ-------------------------------------------------------- 547
EgrARF6B    PSYNDQRQQQQQHQQQPQQS---------------------------------------------QQFNHTSLQQQMPNIITTLPQYGSIGQSQSSSLP-------------------------------------------AISQSQQN 579
AtARF6      QQQQLSQQQQQQLSQQQQQQAYLGVPETHQPQSQAQSQSNNHLSQQQQQVVDNHNPSASSAAVVSAMSQFGSASQPNTSPLQSMTSLCHQQSFSDTNGG--------------------------------------------------- 615
EgrARF6A    PSQFALASQSHTASLQTMPS-----------------------------------------------------------------------------------------------------------------------------LCQQL 575
EgrARF19B   PDQSSSQLPMPDNQIQLQMIQKLQQQQQQQQSLLAQQSALQQPTHPQQLQEQRQHPDVSQSFSKSMVTSQMLEMPQSTANSFSQPNIIPQQIQKNNSQLNVRFSNLSQQPKFQQEQSGLLPEMVGHMGLPQISTSHASASASMLTGAAAG 753
AtARF19     PNQPTGFSQSQLQQQSMLPTGAKMTHQNINSMGNKGLSQMTSFAQEMQFQQQLEMHNSSQLLRNQQEQSSLHSLQQNLSQNPQQLQMQQQSSKPSPSQQLQLQLLQKLQQQQQQQSIPPVSSSLQPQLSALQQTQSHQLQQLLSSQNQQP 696
AtARF7      PLQSHSHPQPQQLQQHKLQQLQVPQNQLYNGQQAAQQHQSQQASTHHLQPQLVSGSMASSVITPPSSSLNQSFQQQQQQSKQLQQAHHHLGASTSQSSVIETSKSSSNLMSAPPQETQFSRQVEQQQPPGLNGQNQQTLLQQKAHQAQAQ 736
EgrARF19A   PLGNGGVVTSNQFPSQMVQQSALYSQLQQQQQQQQQQLLTTQNAPSQPSILGINKVPVHPTSVSQDSKFQSQMEQQQPSLLQRHQQQPMQMQPSSLLQQ---------------SWTQRAQPQLQPQLPQVLQQNLSEQQIQLQLQKLQQ 702
EgrARF24    D----------------------------------------------------------------------------------------------------------------------------------------------------- 446
EgrARF2B    NTWVPQLVQEATLINLVPDS----------------------------------------------------GTFHHRHGIFSSFLRRDSEITKRAKKQ-----------------------------------------NSAEETKTDL 516
AtARF2      ENWMSSARHEPTYTDLLSGF-----------------------------------------------GTNIDPSHGQRIPFYDHSSSPSMPAKRILSDSEGKFDY--------------------------------------------- 553
EgrARF2A    VSHGFSLSSEQSSAANPARK----------------------------------------------------------------------------------------------------------------------HLVDQGRKFHII 522
AtARF1      KSFGVSIGSAF------------------------------------------------------------------------------------------------------------------------------------------- 426
EgrARF1     GSPAALSGYTVN------------------------------------------------------------------------------------------------------------------------------------------ 445
AtARF11     ------------------------------------------------------------------------------------------------------------------------------------------------------ 435
AtARF18     P----------------------------------------------------------------------------------------------------------------------------------------------------- 412
AtARF9      SV---------------------------------------------------------------------------------------------------------------------------------------------------- 423
EgrARF9A    PQTEGDRQFSPSVC---------------------------------------------------------------------------------------------------------------------------------------- 437
EgrARF9B    GGWLPSPCLSVSPNILPDAV--------------------------------------------------------------------------------------------------------------------------DDSKSVSA 460
AtARF13     S----------------------------------------------------------------------------------------------------------------------------------------------------- 424
AtARF14     P----------------------------------------------------------------------------------------------------------------------------------------------------- 428
AtARF23     ------------------------------------------------------------------------------------------------------------------------------------------------------ 222
AtARF15     P----------------------------------------------------------------------------------------------------------------------------------------------------- 433
AtARF20     P----------------------------------------------------------------------------------------------------------------------------------------------------- 412
AtARF21     P----------------------------------------------------------------------------------------------------------------------------------------------------- 428
AtARF12     P----------------------------------------------------------------------------------------------------------------------------------------------------- 428
AtARF22     P----------------------------------------------------------------------------------------------------------------------------------------------------- 426
AtARF4      RFPRVLQGQEICSLKSFPQF----------------------------------------------------------------------------------------------------------------------------AGFSPA 547
EgrARF4     RFPRVLQGQEICTLKSLTTK-----------------------------------------------------------PEYNLGTWGKSSLSCSSFG---------------------------------------------------- 548
AtARF3      GFHKVLQGQE-------------------------------------------------------------------------------------------------------------------------------------------- 483
EgrARF3     GFQMVPSRNGCSALSQGSDN---------------------------------------------------------------------------------------------------------------------------------- 490
AtARF16     P----------------------------------------------------------------------------------------------------------------------------------------------------- 473
EgrARF16B   HSIRSMRTSNGPRMH--------------------------------------------------------------------------------------------------------------------------------------- 504
EgrARF16A   PLNHTSVSTRFSRGAFFG------------------------------------------------------------------------------------------------------------------------------------ 501
AtARF10     LNLSSYTGNNKLH----------------------------------------------------------------------------------------------------------------------------------------- 478
EgrARF10    PHSRSSNSITKDNTQ--------------------------------------------------------------------------------------------------------------------------------------- 498
AtARF17     PP---------------------------------------------------------------------------------------------------------------------------------------------------- 463
EgrARF17    ------------------------------------------------------------------------------------------------------------------------------------------------------ 461
            .......760.......770.......780.......790.......800.......810.......820.......830.......840.......850.......860.......870.......880.......890.......900
```

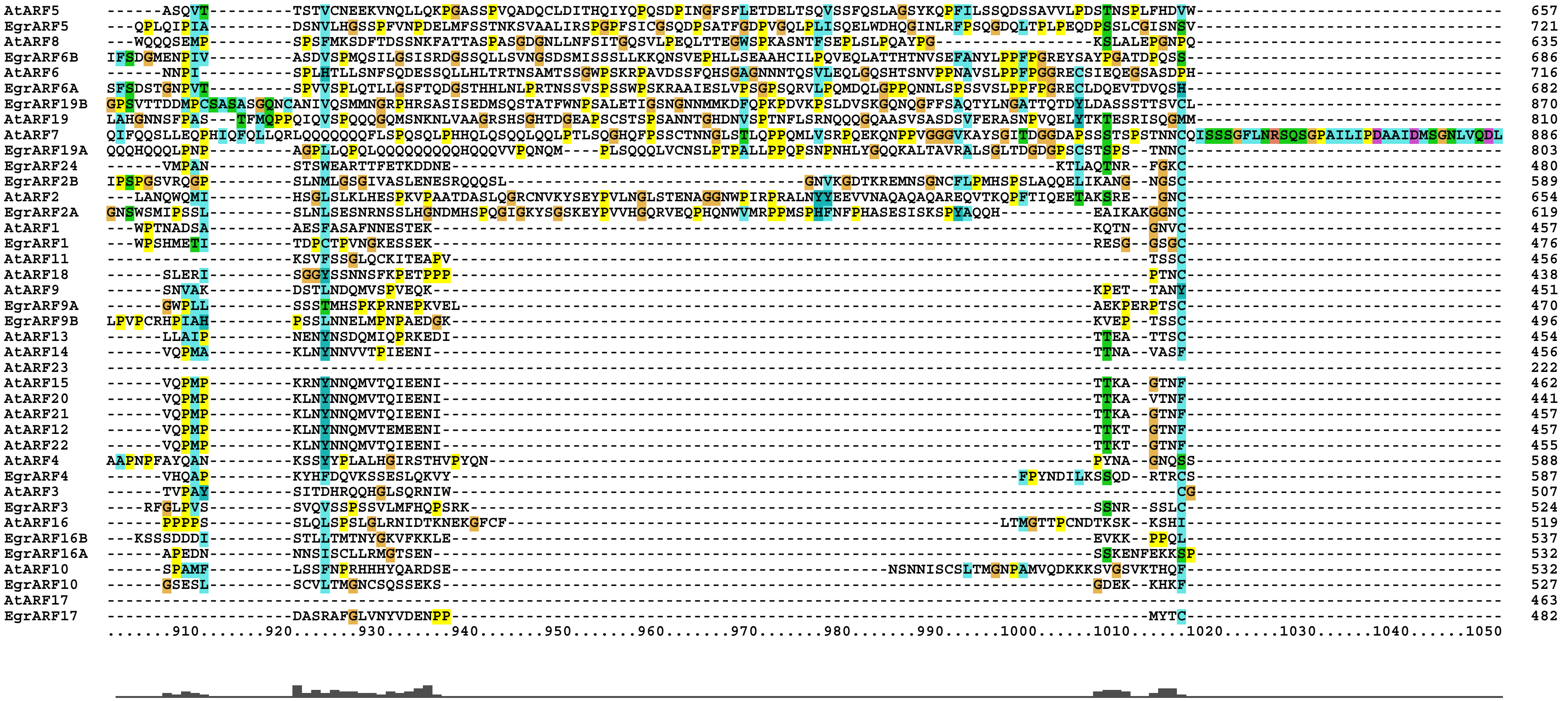

AtARF5 ------ASQVT---------TSTVCNEEKVNQLLQKPGASSPVQADQCLDITHQIYQPQSDPINGFSFLETDELTSQVSSFQSLAGSYKQPFILSSQDSSAVVLPDSTNSPLFHDVW------------------------------ 657
EgrARF5 ---QPLQIPIA---------DSNVLHGSSPFVNPDELMFSSTNKSVAALIRSPGPFSICGSQDPSATFGDPVGQLPLISQELWDHQGINLRFPSQGDQLTPLPEQDPSSLCGISNSV------------------------------ 721
AtARF8 ---WQQQSEMP---------SPSFMKSDFTDSSNKFATTASPASGDGNLLNFSITGQSVLPEQLTTEGWSPKASNTFSEPLSLPQAYPG-----------------KSLALEPGNPQ------------------------------ 635
EgrARF6B IFSDGMENPIV---------ASDVSPMQSILGSISRDGSSQLLSVNGSDSMISSSLLKKQNSVEPHLLSEAAHCILPQVEQLATTHTNVSEFANYLPPFPGREYSAYPGATDPQSS------------------------------ 686
AtARF6 ------NNPI----------SPLHTLLSNFSQDESSQLLHLTRTNSAMTSSGWPSKRPAVDSSFQHSGAGNNNTQSVLEQLGQSHTSNVPPNAVSLPPFPGGRECSIEQEGSASDPH------------------------------ 716
EgrARF6A SFSDSTGNPVT---------SPVVSPLQTLLGSFTQDGSTHHLNLPRTNSSVSPSSWPSKRAAIESLVPSGPSQRVLPQMDQLGPPQNNLSPSSVSLPPFPGRECLDQEVTDVQSH------------------------------ 682
EgrARF19B GPSVTTDDMPCSASASGQNCANIVQSMMNGRPHRSASISEDMSQSTATFWNPSALETIGSNGNNMMKDFQPKPDVKPSLDVSKGQNQGFFSAQTYLNGATTQTDYLDASSSTTSVCL------------------------------ 870
AtARF19 LAHGNNSFPAS---TFMQPPQIQVSPQQQGQMSNKNLVAAGRSHSGHTDGEAPSCSTSPSANNTGHDNVSPTNFLSRNQQQGQAASVSASDSVFERASNPVQELYTKTESRISQGMM------------------------------ 810
AtARF7 QIFQQSLLEQPHIQFQLLQRLQQQQQQQFLSPQSQLPHHQLQSQQLQQLPTLSQGHQFPSSCTNNGLSTLQPPQMLVSRPQEKQNPPVGGGVKAYSGITDGGDAPSSSTSPSTNNCQISSSGFLNRSQSGPAILIPDAAIDMSGNLVQDL 886
EgrARF19A QQQHQQQLPNP---------AGPLLQPQLQQQQQQQQHQQQVVPQNQM----PLSQQQLVCNSLPTPALLPPQPSNPNHLYGQQKALTAVRALSGLTDGDGPSCSTSPS--TNNC------------------------------ 803
EgrARF24 ------VMPAN---------STSWEARTTFETKDDNE-----------------------------------------------------------KTLAQTNR--FGKC------------------------------ 480
EgrARF2B IPSPGSVRQGP---------SLNMLGSGIVASLENESRQQQSL------------------------------GNVKGDTKREMNSGNCFLPMHSPSLAQQELIKANG--NGSC------------------------------ 589
AtARF2 ---LANQWQMI---------HSGLSLKLHESPKVPAATDASLQGRCNVKYSEYPVLNGLSTENAGGNWPIRPRALNYYEEVVNAQAQAQAREQVTKQPFTIQEETAKSRE---GNC------------------------------ 654
EgrARF2A GNSWSMIPSSL---------SLNLSESNRNSSLHGNDMHSPQGIGKYSGSKEYPVVHGQRVEQPHQNWVMRPPMSPHFNFPHASESISKSPYAQQH--------EAIKAKGGNC------------------------------ 619
AtARF1 ---WPTNADSA---------AESFASAFNNESTEK-------------------------------------------------------------KQTN--GNVC------------------------------ 457
EgrARF1 ---WPSHMETI---------TDPCTPVNGKESSEK-------------------------------------------------------------RESG--GSGC------------------------------ 476
AtARF11 --------------------KSVFSSGLQCKITEAPV------------------------------------------------------------------TSSC------------------------------ 456
AtARF18 ------SLERI---------SGGYSSNNSFKPETPPP------------------------------------------------------------------PTNC------------------------------ 438
AtARF9 ------SNVAK---------DSTLNDQMVSPVEQK-------------------------------------------------------------KPET--TANY------------------------------ 451
EgrARF9A ------GWPLL---------SSSTMHSPKPRNEPKVEL----------------------------------------------------------AEKPERPTSC------------------------------ 470
EgrARF9B LPVPCRHPIAH---------PSSLNNELMPNPAEDGK-----------------------------------------------------------KVEP--TSSC------------------------------ 496
AtARF13 ------LLAIP---------NENYNSDQMIQPRKEDI-----------------------------------------------------------TTEA--TTSC------------------------------ 454
AtARF14 ------VQPMA---------KLNYNNVVTPIEENI-------------------------------------------------------------TTNA--VASF------------------------------ 456
AtARF23 -------------------------------------------------------------------------------------------------------------------------------------------------- 222
AtARF15 ------VQPMP---------KRNYNNQMVTQIEENI------------------------------------------------------------TTKA--GTNF------------------------------ 462
AtARF20 ------VQPMP---------KLNYNNQMVTQIEENI------------------------------------------------------------TTKA--VTNF------------------------------ 441
AtARF21 ------VQPMP---------KLNYNNQMVTQIEENI------------------------------------------------------------TTKA--GTNF------------------------------ 457
AtARF12 ------VQPMP---------KLNYNNQMVTEMEENI------------------------------------------------------------TTKT--GTNF------------------------------ 457
AtARF22 ------VQPMP---------KLNYNNQMVTQIEENI------------------------------------------------------------TTKT--GTNF------------------------------ 455
AtARF4 AAPNPFAYQAN---------KSSYYPLALHGIRSTHVPYQN-------------------------------------------------------PYNA--GNQSS------------------------------ 588
EgrARF4 ------VHQAP---------KYHFDQVKSSESLQKVY----------------------------------------------FPYNDILKSSQD--RTRCS------------------------------ 587
AtARF3 ------TVPAY---------SITDHRQHGLSQRNIW----------------------------------------------------------------------CG----------------------------- 507
EgrARF3 ----RFGLPVS---------SVQVSSPSSVLMFHQPSRK---------------------------------------------------------SSNR--SSLC------------------------------ 524
AtARF16 ------PPPPS---------SLQLSPSLGLRNIDTKNEKGFCF------------------------------------------------LTMGTTPCNDTKSK--KSHI------------------------------ 519
EgrARF16B ---KSSSDDDI---------STLLTMTNYGKVFKKLE-----------------------------------------------------------EVKK--PPQL------------------------------ 537
EgrARF16A ------APEDN---------NNSISCLLRMGTSEN-------------------------------------------------------------SSKENFEKKSP------------------------------ 532
AtARF10 ------SPAMF---------LSSFNPRHHHYQARDSE--------------------------------------NSNNISCSLTMGNPAMVQDKKKSVGSVKTHQF------------------------------ 532
EgrARF10 ------GSESL---------SCVLTMGNCSQSSEKS------------------------------------------------------------GDEK--KHKF------------------------------ 527
AtARF17 -------------------------------------------------------------------------------------------------------------------------------------------------- 463
EgrARF17 --------------------DASRAFGLVNYVDENPP-----------------------------------------------------------------MYTC------------------------------ 482
.......910.......920.......930.......940.......950.......960.......970.......980.......990.......1000.......1010.......1020.......1030.......1040.......1050

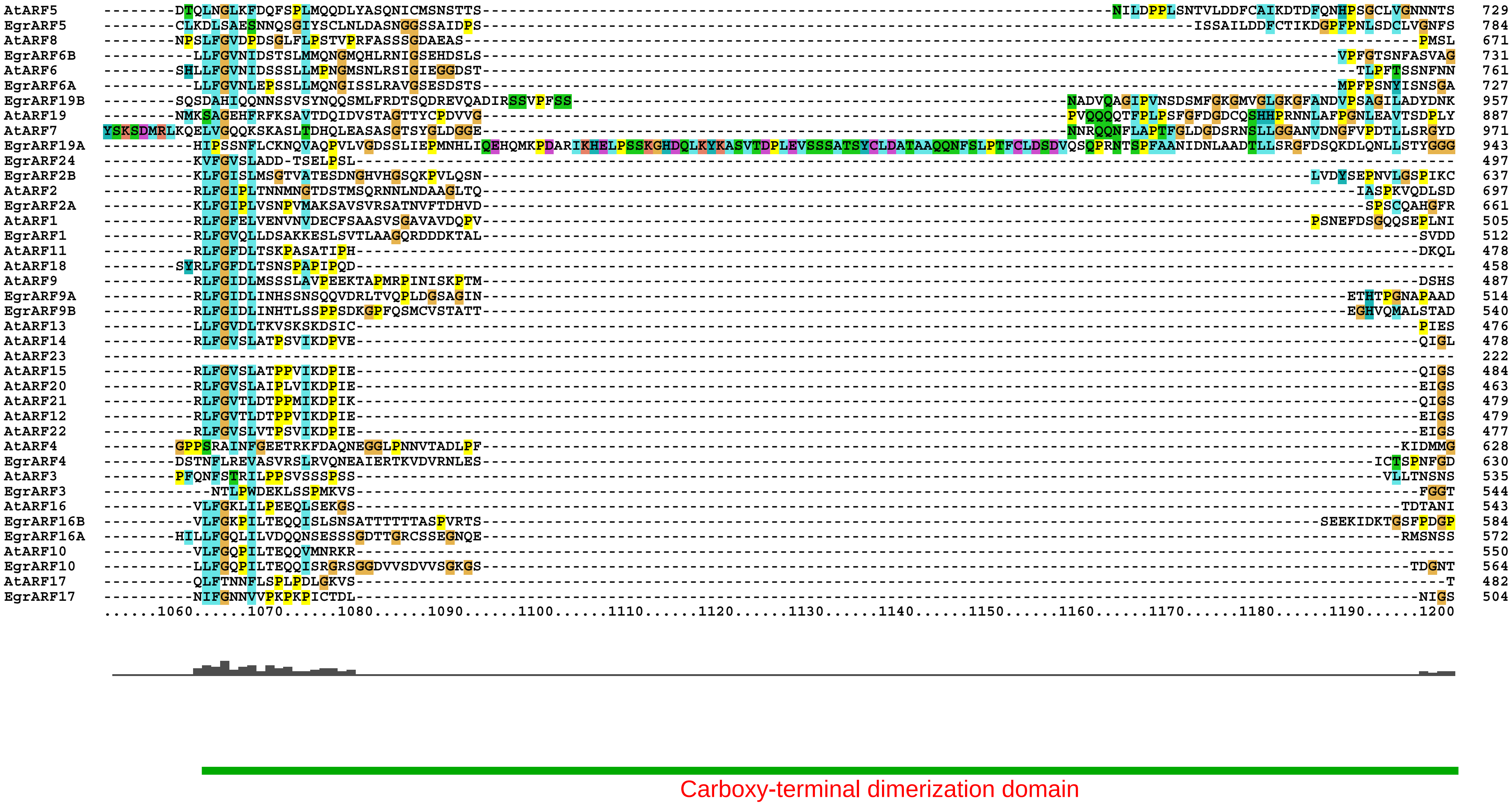

AtARF5 --------DTQLNGLKFDQFSPLMQQDLYASQNICMSNSTTS---NILDPPLSNTVLDDFCAIKDTDFQNHPSGCLVGNNNTS 729
EgrARF5 --------CLKDLSAESNNQSGIYSCLNLDASNGGSSAIDPS---ISSAILDDFCTIKDGPFPNLSDCLVGNFS 784
AtARF8 --------NPSLFGVDPDSGLFLPSTVPRFASSSGDAEAS---PMSL 671
EgrARF6B ----------LLFGVNIDSTSLMMQNGMQHLRNIGSEHDSLS---VPFGTSNFASVAG 731
AtARF6 --------SHLLFGVNIDSSSLLMPNGMSNLRSIGIEGGDST---TLPFTSSNFNN 761
EgrARF6A ----------LLFGVNLEPSSLLMQNGISSLRAVGSESDSTS---MPFPSNYISNSGA 727
EgrARF19B --------SQSDAHIQQNNSSVSYNQQSMLFRDTSQDREVQADIRSSVPFSS---NADVQAGIPVNSDSMFGKGMVGLGKGFANDVPSAGILADYDNK 957
AtARF19 --------NMKSAGEHFRFKSAVTDQIDVSTAGTTYCPDVVG---PVQQQQTFPLPSFGFDGDCQSHHPRNNLAFPGNLEAVTSDPLY 887
AtARF7 YSKSDMRLKQELVGQQKSKASLTDHQLEASASGTSYGLDGGE---NNRQQNFLAPTFGLDGDSRNSLLGGANVDNGFVPDTLLSRGYD 971
EgrARF19A --------HIPSSNFLCKNQVAQPVLVGDSSLIEPMNHLIQEHQMKPDARIKHELPSSKGHDQLKYKASVTDPLEVSSSATSYCLDATAAQQNFSLPTFCLDSDVQSQPRNTSPFAANIDNLAADTLLSRGFDSQKDLQNLLSTYGGG 943
EgrARF24 ----------KVFGVSLADD-TSELPSL--- 497
EgrARF2B ----------KLFGISLMSGTVATESDNGHVHGSQKPVLQSN---LVDYSEPNVLGSPIKC 637
AtARF2 ----------RLFGIPLTNNMNGTDSTMSQRNNLNDAAGLTQ---IASPKVQDLSD 697
EgrARF2A ----------KLFGIPLVSNPVMAKSAVSVRSATNVFTDHVD---SPSCQAHGFR 661
AtARF1 ----------RLFGFELVENVNVDECFSAASVSGAVAVDQPV---PSNEFDSGQQSEPLNI 505
EgrARF1 ----------RLFGVQLLDSAKKESLSVTLAAGQRDDDKTAL---SVDD 512
AtARF11 ----------RLFGFDLTSKPASATIPH---DKQL 478
AtARF18 --------SYRLFGFDLTSNSPAPIPQD--- 458
AtARF9 ----------RLFGIDLMSSSLAVPEEKTAPMRPINISKPTM---DSHS 487
EgrARF9A ----------RLFGIDLINHSSNSQQVDRLTVQPLDGSAGIN---ETHTPGNAPAAD 514
EgrARF9B ----------RLFGIDLINHTLSSPPSDKGPFQSMCVSTATT---EGHVQMALSTAD 540
AtARF13 ----------LLFGVDLTKVSKSKDSIC---PIES 476
AtARF14 ----------RLFGVSLATPSVIKDPVE---QIGL 478
AtARF23 --- 222
AtARF15 ----------RLFGVSLATPPVIKDPIE---QIGS 484
AtARF20 ----------RLFGVSLAIPLVIKDPIE---EIGS 463
AtARF21 ----------RLFGVTLDTPPMIKDPIK---QIGS 479
AtARF12 ----------RLFGVTLDTPPVIKDPIE---EIGS 479
AtARF22 ----------RLFGVSLVTPSVIKDPIE---EIGS 477
AtARF4 --------GPPSRAINFGEETRKFDAQNEGGLPNNVTADLPF---KIDMMG 628
EgrARF4 --------DSTNFLREVASVRSLRVQNEAIERTKVDVRNLES---ICTSPNFGD 630
AtARF3 --------PFQNFSTRILPPSVSSSPSS---VLLTNSNS 535
EgrARF3 ------------NTLPWDEKLSSPMKVS---FGGT 544
AtARF16 ----------VLFGKLILPEEQLSEKGS---TDTANI 543
EgrARF16B ----------VLFGKPILTEQQISLSNSATTTTTASPVRTS---SEEKIDKTGSFPDGP 584
EgrARF16A --------HILLFGQLILVDQQNSESSSGDTTGRCSSEGNQE---RMSNSS 572
AtARF10 ----------VLFGQPILTEQQVMNRKR--- 550
EgrARF10 ----------LLFGQPILTEQQISRGRSGGDVVSDVVSGKGS---TDGNT 564
AtARF17 ----------QLFTNNFLSPLPDLGKVS---T 482
EgrARF17 ----------NIFGNNVVPKPKPICTDL---NIGS 504
.....1060.....1070.....1080.....1090.....1100.....1110.....1120.....1130.....1140.....1150.....1160.....1170.....1180.....1190.....1200
Carboxy-terminal dimerization domain

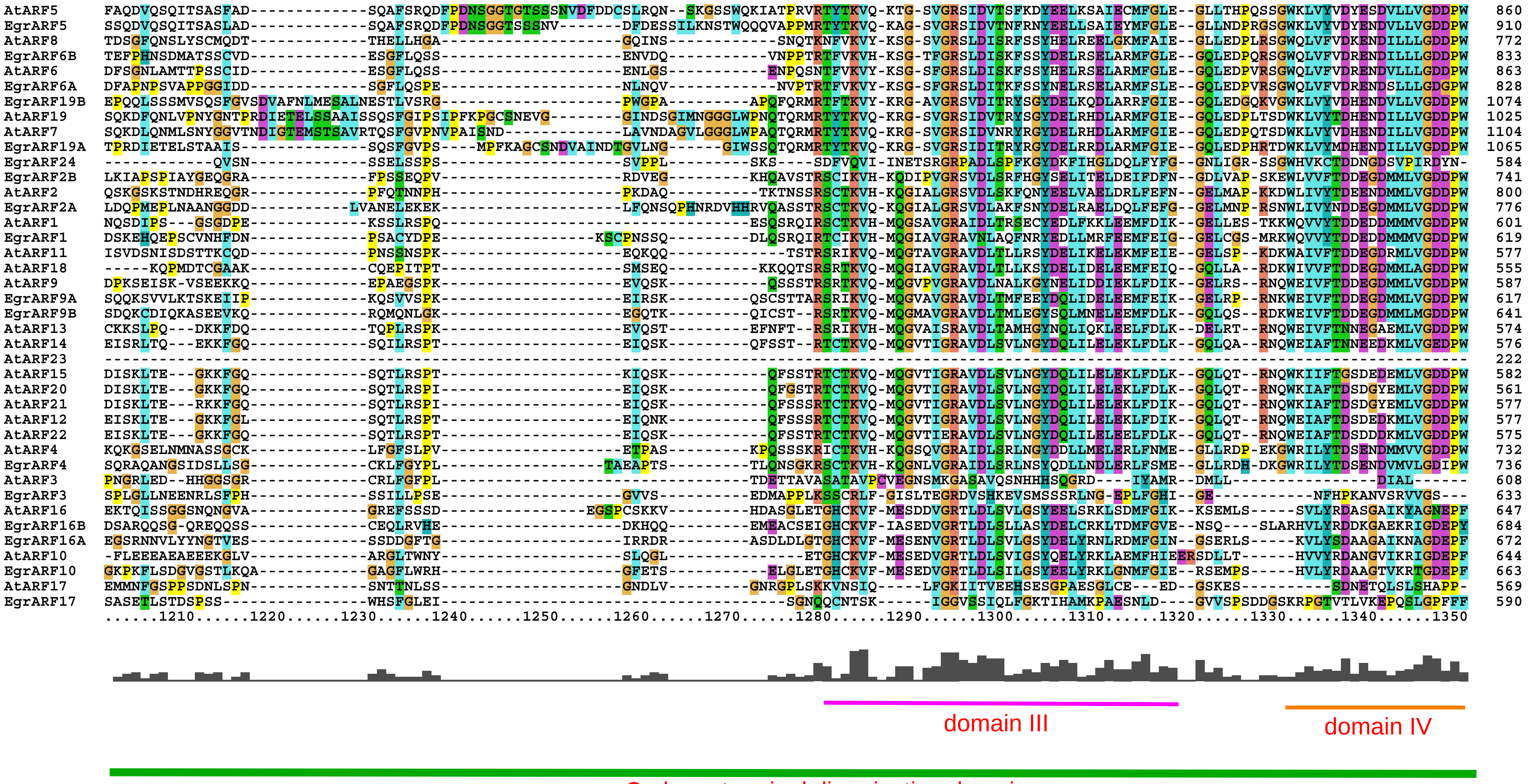
domain III
domain IV
Carboxy-terminal dimerization domain

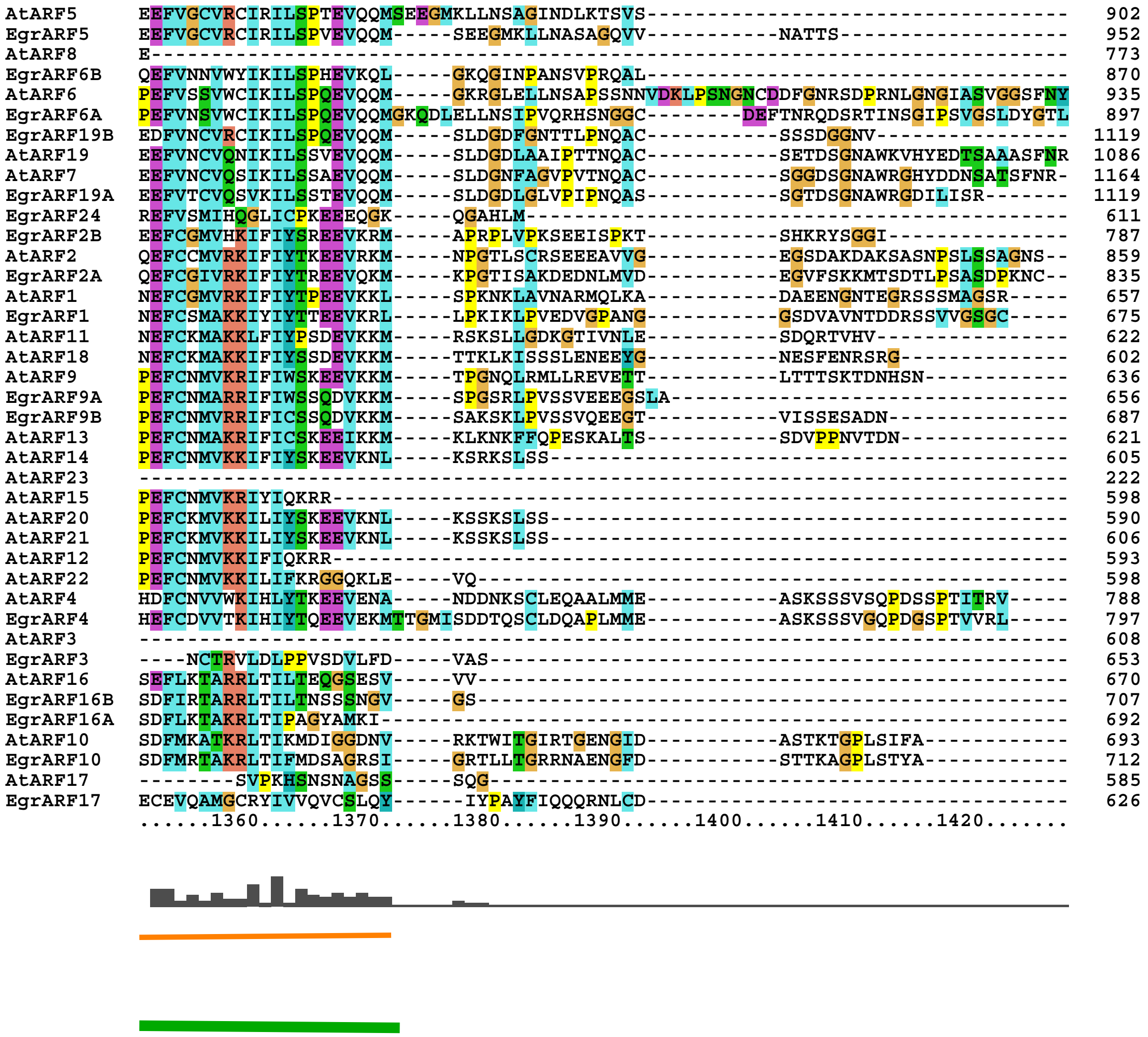
AtARF5 EEFVGCVRCIRILSPTEVQQMSEEGMKLLNSAGINDLKTSVS----------------------------------- 902
EgrARF5 EEFVGCVRCIRILSPVEVQQM-----SEEGMKLLNASAGQVV-----------NATTS------------------- 952
AtARF8 E---------------------------------------------------------------------------- 773
EgrARF6B QEFVNNVWYIKILSPHEVKQL-----GKQGINPANSVPRQAL----------------------------------- 870
AtARF6 PEFVSSVWCIKILSPQEVQQM-----GKRGLELLNSAPSSNNVDKLPSNGNCDDFGNRSDPRNLGNGIASVGGSFNY 935
EgrARF6A PEFVNSVWCIKILSPQEVQQMGKQDLELLNSIPVQRHSNGGC--------DEFTNRQDSRTINSGIPSVGSLDYGTL 897
EgrARF19B EDFVNCVRCIKILSPQEVQQM-----SLDGDFGNTTLPNQAC-----------SSSDGGNV---------------- 1119
AtARF19 EEFVNCVQNIKILSSVEVQQM-----SLDGDLAAIPTTNQAC-----------SETDSGNAWKVHYEDTSAAASFNR 1086
AtARF7 EEFVNCVQSIKILSSAEVQQM-----SLDGNFAGVPVTNQAC-----------SGGDSGNAWRGHYDDNSATSFNR- 1164
EgrARF19A EEFVTCVQSVKILSSTEVQQM-----SLDGDLGLVPIPNQAS-----------SGTDSGNAWRGDILISR------- 1119
EgrARF24 REFVSMIHQGLICPKEEEQGK-----QGAHLM--------------------------------------------- 611
EgrARF2B EEFCGMVHKIFIYSREEVKRM-----APRPLVPKSEEISPKT-----------SHKRYSGGI--------------- 787
AtARF2 QEFCCMVRKIFIYTKEEVRKM-----NPGTLSCRSEEEAVVG-----------EGSDAKDAKSASNPSLSSAGNS-- 859
EgrARF2A QEFCGIVRKIFIYTREEVQKM-----KPGTISAKDEDNLMVD-----------EGVFSKKMTSDTLPSASDPKNC-- 835
AtARF1 NEFCGMVRKIFIYTPEEVKKL-----SPKNKLAVNARMQLKA-----------DAEENGNTEGRSSSMAGSR----- 657
EgrARF1 NEFCSMAKKIYIYTTEEVKRL-----LPKIKLPVEDVGPANG-----------GSDVAVNTDDRSSVVGSGC----- 675
AtARF11 NEFCKMAKKLFIYPSDEVKKM-----RSKSLLGDKGTIVNLE-----------SDQRTVHV---------------- 622
AtARF18 NEFCKMAKKIFIYSSDEVKKM-----TTKLKISSSLENEEYG-----------NESFENRSRG-------------- 602
AtARF9 PEFCNMVKRIFIWSKEEVKKM-----TPGNQLRMLLREVETT-----------LTTTSKTDNHSN------------ 636
EgrARF9A PEFCNMARRIFIWSSQDVKKM-----SPGSRLPVSSVEEEGSLA--------------------------------- 656
EgrARF9B PEFCNMVRRIFICSSQDVKKM-----SAKSKLPVSSVQEEGT-----------VISSESADN--------------- 687
AtARF13 PEFCNMAKRIFICSKEEIKKM-----KLKNKFFQPESKALTS-----------SDVPPNVTDN-------------- 621
AtARF14 PEFCNMVKKIFIYSKEEVKNL-----KSRKSLSS------------------------------------------- 605
AtARF23 ----------------------------------------------------------------------------- 222
AtARF15 PEFCNMVKRIYIQKRR------------------------------------------------------------- 598
AtARF20 PEFCKMVKKILIYSKEEVKNL-----KSSKSLSS------------------------------------------- 590
AtARF21 PEFCKMVKKILIYSKEEVKNL-----KSSKSLSS------------------------------------------- 606
AtARF12 PEFCNMVKKIFIQKRR------------------------------------------------------------- 593
AtARF22 PEFCNMVKKILIFKRGGQKLE-----VQ------------------------------------------------- 598
AtARF4 HDFCNVVWKIHLYTKEEVENA-----NDDNKSCLEQAALMME-----------ASKSSSVSQPDSSPTITRV----- 788
EgrARF4 HEFCDVVTKIHIYTQEEVEKMTTGMISDDTQSCLDQAPLMME-----------ASKSSSVGQPDGSPTVVRL----- 797
AtARF3 ----------------------------------------------------------------------------- 608
EgrARF3 ----NCTRVLDLPPVSDVLFD-----VAS------------------------------------------------ 653
AtARF16 SEFLKTARRLTILTEQGSESV-----VV------------------------------------------------- 670
EgrARF16B SDFIRTARRLTILTNSSSNGV-----GS------------------------------------------------- 707
EgrARF16A SDFLKTAKRLTIPAGYAMKI--------------------------------------------------------- 692
AtARF10 SDFMKATKRLTIKMDIGGDNV-----RKTWITGIRTGENGID-----------ASTKTGPLSIFA------------ 693
EgrARF10 SDFMRTAKRLTIFMDSAGRSI-----GRTLLTGRRNAENGFD-----------STTKAGPLSTYA------------ 712
AtARF17 --------SVPKHSNSNAGSS-----SQG------------------------------------------------ 585
EgrARF17 ECEVQAMGCRYIVVQVCSLQY------IYPAYFIQQRNLCD------------------------------------ 626
......1360......1370......1380......1390......1400......1410......1420.......

**Supplementary Figure 3.** Multiple sequence alignment of predicted amino acid sequences of *Egr*ARF and *At*ARF proteins. The multiple sequence alignment was obtained with muscle and manual correction. The highly conserved domains and nuclear localization signals (NLSs) of ARF proteins were noted on the bottom of the alignment with different colours. The amino acid position was given on the right of each sequence.
