# Supplementary material for: Genome-Wide Characterization and Expression Profiling of the AUXIN RESPONSE FACTOR (ARF) Gene Family in Eucalyptus grandis
Source: PLoS One. 2014 Sep 30;9(9):e108906. doi: 10.1371/journal.pone.0108906 (PMC4182523; doi:10.1371/journal.pone.0108906)

Name	Accession number	Alternative splicing
EgrARF1	Eucgr.G00076.1	
	Eucgr.G00076.2	
	Eucgr.G00076.3	
	Eucgr.G00076.4	
EgrARF2A	Eucgr.K02197.1	
	Eucgr.K02197.2	
	Eucgr.K02197.3	
	Eucgr.K02197.4	
	Eucgr.K02197.5	
EgrARF2B	Eucgr.B03551.1	
	Eucgr.B03551.2	
EgrARF3	Eucgr.D00588.1	
	Eucgr.D00588.2	
EgrARF4	Eucgr.B02480.1	
	Eucgr.B02480.2	
	Eucgr.B02480.3	
EgrARF5	Eucgr.F02090.1	
EgrARF6A	Eucgr.D00264.1	
	Eucgr.D00264.2	
EgrARF6B	Eucgr.A02065.1	
	Eucgr.A02065.2	
	Eucgr.A02065.3	
EgrARF9A	Eucgr.D01764.1	
EgrARF9B	Eucgr.E00888.1	
	Eucgr.E00888.2	
EgrARF10	Eucgr.J00923.1	
EgrARF16A	Eucgr.G02838.1	
EgrARF16B	Eucgr.K01240.1	
EgrARF17	Eucgr.F04380.1	
EgrARF19A	Eucgr.C03293.1	
	Eucgr.C03293.2	
EgrARF19B	Eucgr.C02178.1	
	Eucgr.C02178.2	
EgrARF24	Eucgr.K03433.1	

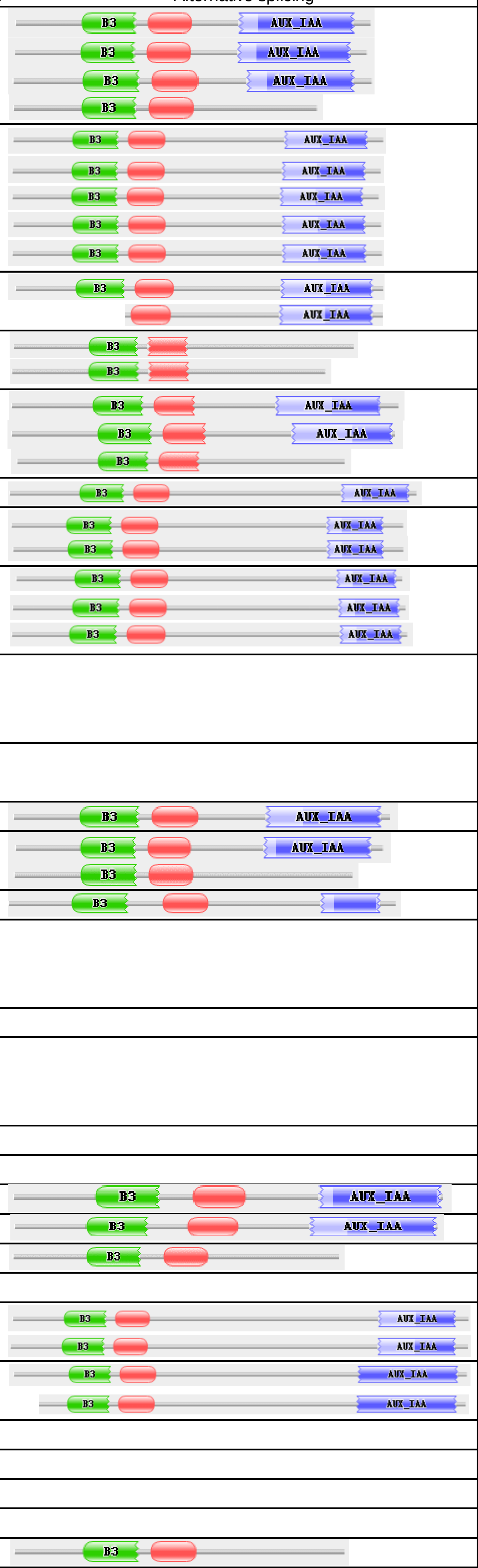

Name	Accession number	Alternative splicing
AtARF1	AT1G59750.1	
	AT1G59750.2	
	AT1G59750.3	
	AT1G59750.4	
AtARF2	AT5G62000.1	
	AT5G62000.2	
	AT5G62000.3	
	AT5G62000.4	
AtARF3	AT2G33860	
AtARF4	AT5G60450	
	Δ AtARF4	
AtARF5	AT1G19850	
AtARF6	AT1G30330	
AtARF7	AT5G20730.1	
	AT5G20730.2	
	AT5G20730.3	
AtARF8	AT5G37020.1	
	AT5G37020.2	
AtARF9	AT4G23980.1	
	AT4G23980.2	
AtARF10	AT2G28350	
AtARF11	AT2G46530.1	
	AT2G46530.2	
	AT2G46530.3	
AtARF12	AT1G34310	
AtARF13	AT1G34170.1	
	AT1G34170.2	
	AT1G34170.3	
AtARF14	AT1G35540	
AtARF15	AT1G35520	
AtARF16	AT4G30080	
AtARF17	AT1G77850	
AtARF18	AT3G61830	
AtARF19	AT1G19220	
AtARF20	AT1G35240	
AtARF21	AT1G34410	
AtARF22	AT1G34390	
AtARF23	AT1G43950	

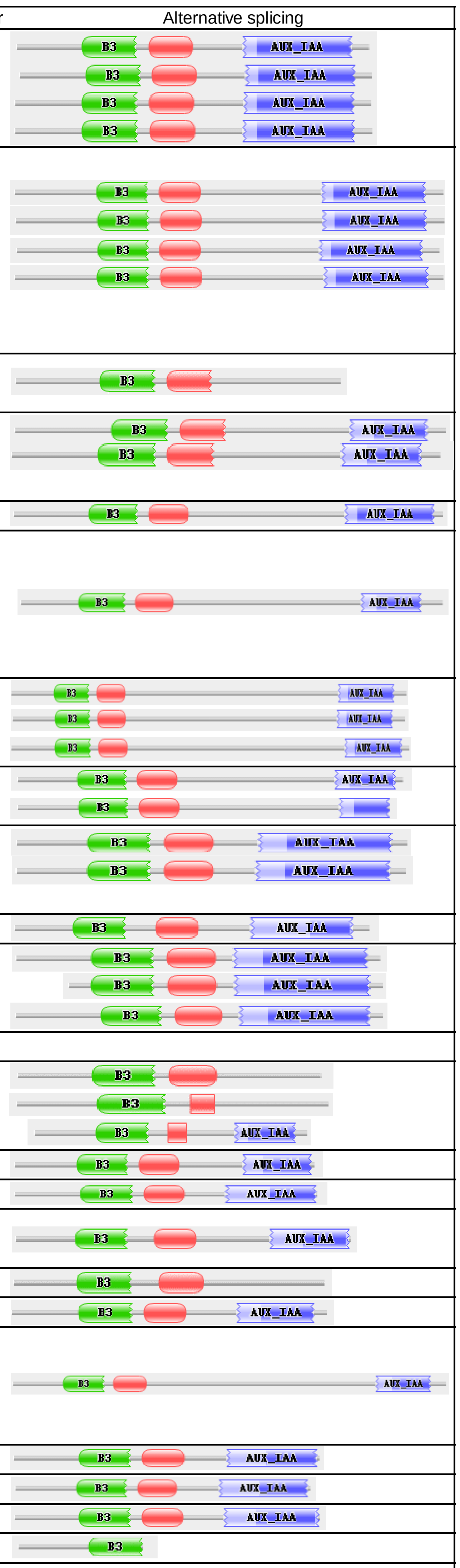

Supplement: Figure S4 — Comparative analysis of predicted ARF alternative variants between Eucalyptus grandis and Arabidopsis thaliana . The alternative spliced protein sequences were extracted from Phytozome except for AtARF4 (obtained from Finet et al. (2013), the motif structures were predicted by Pfam (http://pfam.xfam.org/). (PDF) [file pone.0108906.s004.pdf]
