# Supplementary material for: Genome-Wide Characterization and Expression Profiling of the AUXIN RESPONSE FACTOR (ARF) Gene Family in Eucalyptus grandis
Source: PLoS One. 2014 Sep 30;9(9):e108906. doi: 10.1371/journal.pone.0108906 (PMC4182523; doi:10.1371/journal.pone.0108906)
Supplement: Figure S6 — Comparative Phylogenetic relationships between ARF proteins from poplar, Eucalyptus , grapevine, Arabidopsis , tomato and rice. Full-length protein sequences were aligned using the Clustal_X program. The phylogenetic tree was constructed by using the MEGA5 program and the neighbour-joining method with predicted full-length ARF proteins. Bootstrap supports are indicated at each node. (PDF) [file pone.0108906.s006.pdf]

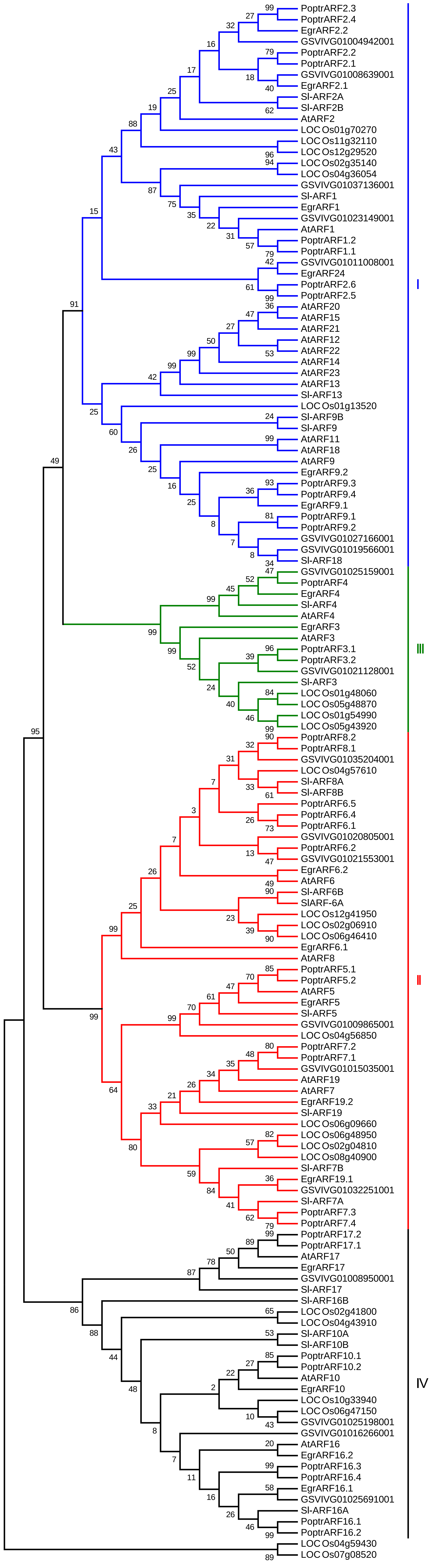
PoptrARF2.3
PoptrARF2.4
EgrARF2.2
GSVIVG01004942001
PoptrARF2.2
PoptrARF2.1
GSVIVG01008639001
EgrARF2.1
Sl-ARF2A
Sl-ARF2B
AtARF2
LOC Os01g70270
LOC Os11g32110
LOC Os12g29520
LOC Os02g35140
LOC Os04g36054
GSVIVG01037136001
Sl-ARF1
EgrARF1
GSVIVG01023149001
AtARF1
PoptrARF1.2
PoptrARF1.1
GSVIVG01011008001
EgrARF24
PoptrARF2.6
PoptrARF2.5
AtARF20
AtARF15
AtARF21
AtARF12
AtARF22
AtARF14
AtARF23
AtARF13
Sl-ARF13
LOC Os01g13520
Sl-ARF9B
Sl-ARF9
AtARF11
AtARF18
AtARF9
EgrARF9.2
PoptrARF9.3
PoptrARF9.4
EgrARF9.1
PoptrARF9.1
PoptrARF9.2
GSVIVG01027166001
GSVIVG01019566001
Sl-ARF18
I
GSVIVG01025159001
PoptrARF4
EgrARF4
Sl-ARF4
AtARF4
EgrARF3
AtARF3
PoptrARF3.1
PoptrARF3.2
GSVIVG01021128001
Sl-ARF3
LOC Os01g48060
LOC Os05g48870
LOC Os01g54990
LOC Os05g43920
III
PoptrARF8.2
PoptrARF8.1
GSVIVG01035204001
LOC Os04g57610
Sl-ARF8A
Sl-ARF8B
PoptrARF6.5
PoptrARF6.4
PoptrARF6.1
GSVIVG01020805001
PoptrARF6.2
GSVIVG01021553001
EgrARF6.2
AtARF6
Sl-ARF6B
SlARF-6A
LOC Os12g41950
LOC Os02g06910
LOC Os06g46410
EgrARF6.1
AtARF8
PoptrARF5.1
PoptrARF5.2
AtARF5
EgrARF5
Sl-ARF5
GSVIVG01009865001
LOC Os04g56850
PoptrARF7.2
PoptrARF7.1
GSVIVG01015035001
AtARF19
AtARF7
EgrARF19.2
Sl-ARF19
LOC Os06g09660
LOC Os06g48950
LOC Os02g04810
LOC Os08g40900
Sl-ARF7B
EgrARF19.1
GSVIVG01032251001
Sl-ARF7A
PoptrARF7.3
PoptrARF7.4
II
PoptrARF17.2
PoptrARF17.1
AtARF17
EgrARF17
GSVIVG01008950001
Sl-ARF17
Sl-ARF16B
LOC Os02g41800
LOC Os04g43910
Sl-ARF10A
Sl-ARF10B
PoptrARF10.1
PoptrARF10.2
AtARF10
EgrARF10
LOC Os10g33940
LOC Os06g47150
GSVIVG01025198001
GSVIVG01016266001
AtARF16
EgrARF16.2
PoptrARF16.3
PoptrARF16.4
EgrARF16.1
GSVIVG01025691001
Sl-ARF16A
PoptrARF16.1
PoptrARF16.2
IV
LOC Os04g59430
LOC Os07g08520

Supplementary Figure 5. Phylogenetic relationships between ARF proteins from woody species (poplar, *Eucalyptus* and grapevine) herbaceous species (*Arabidopsis*, tomato and rice). Full-length protein sequences were aligned by using the Clustal_X program. The phylogenetic tree was constructed by using the MEGA5 program and the neighbor-joining method with predicted full-length ARF proteins. Bootstrap support is indicated at each node.
