# Supplementary material for: Genome-Wide Characterization and Expression Profiling of the AUXIN RESPONSE FACTOR (ARF) Gene Family in Eucalyptus grandis
Source: PLoS One. 2014 Sep 30;9(9):e108906. doi: 10.1371/journal.pone.0108906 (PMC4182523; doi:10.1371/journal.pone.0108906)
Supplement: Table S1 — Primers for EgrARF genes and reference genes used in qRT-PCR experiments. (PDF) [file pone.0108906.s011.pdf]

Table S1. The primers for *EgrARF* genes and reference genes used in qRT-PCR experiments

Name	Accession No.	Forward 5'-3'	Reverse 5'-3'	Efficiency
ARF1	Eucgr.G00076.1	TTTAAGAGCGCTGTGGTTCTCTG	AAGCATCAGCAAACGCACCTTG	1.90
ARF2A	Eucgr.K02197.1	GGCATGCCATTTCTACAGGAACC	TCAGCAGGGCTTATCCTAGGTTTG	1.97
ARF2B	Eucgr.B03551.1	ATTGAGGGCTGAACCAGAGACC	TGGTTAGGCACCGGAAGCAAAG	1.95
ARF3	Eucgr.D00588.1	ACATATATACCGGGCAGGACAGC	TCTTCCGGTTTACAAATGCACTCC	1.94
ARF4	Eucgr.B02480.1	AGCTGTCGCTAGTGCTGTATCC	AACTCTGCATGGCTTGCCCTTG	1.96
ARF5	Eucgr.F02090.1	GCACATGGCAACAGCAAGTAGC	ATCGACCTTCCGACTGATCCTG	1.97
ARF6A	Eucgr.D00264.1	TGAGCTTGCTCGCATGTTTAGCC	TGTTCACAAACTCCGGCCAAGG	1.89
ARF6B	Eucgr.A02065.1	GCTGGCAGCTTGTATTTGTAGACC	CCACACATTGTTGACGAACTCCTG	1.88
ARF9A	Eucgr.D01764.1	TCCAAAGCAGTCTGTGGTTTCACC	ATGCGACTCCTTGCAGTGGTAG	1.87
ARF9B	Eucgr.E00888.1	GTGACTCGTTCGTGTTCTTAAGGG	TGACGAGCAACTCGTTTCACTCC	2.00
ARF10	Eucgr.J00923.1	TAGCAGGGCGTGTCGTGTTATC	AATCCGGACGCTTGCGTTTCTC	1.90
ARF16A	Eucgr.G02838.1	AGGGACATGTTCGGCATAAACGG	AATAGCACCGGCAGCATCTGAG	1.84
ARF16B	Eucgr.K01240.1	CTCAGAAATCGGCCACTGCAAAG	TGAGGCAAGCAACGAGAGATCG	1.97
ARF17	Eucgr.F04380.1	ATGGAGGTGGTGGTGAATGCAG	AAACCACCTCGAACGGCAATCC	1.85
ARF19A	Eucgr.C03293.1	TCAGTTTCAAGCGGATGAGAAGCC	AAGGACAGCCACTCTGGGTTAG	1.78
ARF19B	Eucgr.C02178.1	TCAAACAGGACCTGGCTCGTAG	CGAACACAGTTCACGAAGTCCTC	1.93
ARF24	Eucgr.K03433.1	TCCAGCGACTTTGTGCAGGTTATC	AGCCTTTGAATGGGCTGAGGTC	1.92
EF-1α	Eucgr.B02473.1	ATGCGTCAGACTGTGGCTGTTG	TTGGTCACCTTGGCTCCACTTG	1.84
SAND	Eucgr.B02502.1	TTGATCCACTTGCGGACAAGGC	TCACCCATTGACATACACGATTGC	2.11
PP2A1	Eucgr.B03386.1	TCGAGCTTTGGACCGCATACAAG	ACCACAAGAGGTCACACATTGGC	1.98
PP2A3 5'end	Eucgr.B03031.1	CGGAAGAACTGGGTGTGTTT	CACAGAGGGTCTCCAATGGT	2.03
PP2A3 3'end	Eucgr.B03031.1	CAGCGGCAAACAACTTGAAGCG	ATTATGTGCTGCATTGCCCAGTC	2.02
IDH 5'end	Eucgr.F02901.1	AATCGACCTGCTTCGACCCTTC	TCGACCTTGATCTTCTCGAAACCC	1.92
IDH 3'end	Eucgr.F02901.1	TGCTGTGGCAGCTGAACTCAAG	ATGTTGTCCGCCAGTCACCTAC	1.86
