# Supplementary material for: Genome-Wide Characterization and Expression Profiling of the AUXIN RESPONSE FACTOR (ARF) Gene Family in Eucalyptus grandis
Source: PLoS One. 2014 Sep 30;9(9):e108906. doi: 10.1371/journal.pone.0108906 (PMC4182523; doi:10.1371/journal.pone.0108906)
Supplement: Table S2 — Protein identity matrix between Egr ARF and At ARF. (PDF) [file pone.0108906.s012.pdf]

Table S2. Protein identity matrix between EgrARF and AtARF

	EgrARF1	EgrARF2A	EgrARF2B	EgrARF3	EgrARF4	EgrARF5	EgrARF6A	EgrARF6B	EgrARF9A	EgrARF9B	EgrARF10	EgrARF16A	EgrARF16B	EgrARF17	EgrARF19A	EgrARF19B	EgrARF24
AtARF1	0.622	0.388	0.399	0.239	0.31	0.244	0.251	0.257	0.471	0.446	0.226	0.222	0.216	0.194	0.203	0.208	0.309
AtARF2	0.39	0.593	0.495	0.23	0.297	0.226	0.249	0.246	0.361	0.345	0.193	0.185	0.181	0.164	0.206	0.203	0.268
AtARF3	0.244	0.22	0.242	0.462	0.326	0.192	0.196	0.205	0.247	0.23	0.195	0.197	0.187	0.206	0.153	0.157	0.23
AtARF4	0.298	0.28	0.295	0.31	0.578	0.226	0.231	0.247	0.282	0.286	0.198	0.193	0.195	0.168	0.201	0.202	0.24
AtARF5	0.242	0.247	0.247	0.195	0.224	0.593	0.342	0.349	0.235	0.232	0.181	0.176	0.181	0.156	0.311	0.302	0.2
AtARF6	0.243	0.247	0.244	0.18	0.22	0.337	0.691	0.656	0.238	0.235	0.181	0.168	0.18	0.143	0.369	0.349	0.197
AtARF7	0.196	0.216	0.208	0.147	0.186	0.293	0.32	0.332	0.187	0.183	0.148	0.145	0.147	0.115	0.59	0.415	0.159
AtARF8	0.263	0.235	0.254	0.197	0.236	0.334	0.518	0.541	0.266	0.251	0.191	0.189	0.194	0.166	0.329	0.317	0.214
AtARF9	0.435	0.347	0.367	0.224	0.273	0.232	0.247	0.25	0.586	0.584	0.236	0.218	0.213	0.195	0.208	0.193	0.3
AtARF10	0.221	0.196	0.206	0.184	0.192	0.165	0.183	0.188	0.235	0.229	0.601	0.477	0.493	0.292	0.154	0.158	0.205
AtARF11	0.449	0.356	0.357	0.224	0.271	0.235	0.253	0.25	0.522	0.488	0.238	0.229	0.227	0.204	0.198	0.202	0.341
AtARF12	0.347	0.285	0.298	0.214	0.243	0.193	0.208	0.212	0.397	0.369	0.206	0.214	0.209	0.191	0.169	0.172	0.312
AtARF13	0.344	0.271	0.286	0.2	0.236	0.189	0.204	0.207	0.391	0.374	0.188	0.202	0.192	0.172	0.159	0.164	0.289
AtARF14	0.357	0.285	0.308	0.213	0.247	0.2	0.21	0.218	0.412	0.385	0.2	0.208	0.205	0.187	0.169	0.173	0.317
AtARF15	0.343	0.278	0.29	0.209	0.24	0.193	0.208	0.213	0.393	0.368	0.205	0.217	0.208	0.196	0.167	0.171	0.3
AtARF16	0.214	0.178	0.2	0.197	0.196	0.166	0.185	0.179	0.227	0.217	0.525	0.58	0.525	0.318	0.151	0.152	0.197
AtARF17	0.179	0.149	0.155	0.171	0.141	0.139	0.138	0.139	0.177	0.162	0.284	0.276	0.291	0.421	0.113	0.123	0.167
AtARF18	0.449	0.348	0.354	0.227	0.283	0.225	0.252	0.256	0.519	0.486	0.233	0.229	0.225	0.204	0.201	0.197	0.335
AtARF19	0.207	0.215	0.214	0.161	0.199	0.318	0.346	0.354	0.198	0.195	0.164	0.156	0.16	0.129	0.604	0.448	0.169
AtARF20	0.351	0.283	0.297	0.21	0.249	0.196	0.212	0.218	0.399	0.381	0.204	0.216	0.211	0.196	0.17	0.172	0.309
AtARF21	0.357	0.283	0.295	0.21	0.249	0.198	0.212	0.221	0.398	0.375	0.205	0.214	0.212	0.191	0.175	0.176	0.308
AtARF22	0.356	0.284	0.299	0.211	0.24	0.195	0.208	0.212	0.407	0.382	0.201	0.209	0.202	0.188	0.17	0.173	0.307
AtARF23	0.163	0.13	0.138	0.14	0.119	0.088	0.094	0.098	0.176	0.161	0.106	0.116	0.108	0.127	0.073	0.074	0.155
