# Supplementary material for: Genome-Wide Characterization and Expression Profiling of the AUXIN RESPONSE FACTOR (ARF) Gene Family in Eucalyptus grandis
Source: PLoS One. 2014 Sep 30;9(9):e108906. doi: 10.1371/journal.pone.0108906 (PMC4182523; doi:10.1371/journal.pone.0108906)
Supplement: Table S3 — Protein identity matrix among Egr ARF. (PDF) [file pone.0108906.s013.pdf]

Table S3. Protein identity matrix among EgrARF

	EgrARF1	EgrARF2A	EgrARF2B	EgrARF3	EgrARF4	EgrARF5	EgrARF6A	EgrARF6B	EgrARF9A	EgrARF9B	EgrARF10	EgrARF16A	EgrARF16B	EgrARF17	EgrARF19A	EgrARF19B	EgrARF24
EgrARF1	ID	0.389	0.411	0.246	0.29	0.247	0.261	0.267	0.503	0.471	0.237	0.245	0.233	0.2	0.208	0.206	0.329
EgrARF2A	0.389	ID	0.515	0.222	0.276	0.247	0.261	0.262	0.372	0.371	0.202	0.204	0.206	0.167	0.215	0.218	0.296
EgrARF2B	0.411	0.515	ID	0.233	0.285	0.252	0.265	0.266	0.387	0.388	0.211	0.205	0.204	0.166	0.218	0.218	0.317
EgrARF3	0.246	0.222	0.233	ID	0.363	0.197	0.196	0.2	0.237	0.241	0.21	0.207	0.202	0.18	0.165	0.164	0.226
EgrARF4	0.29	0.276	0.285	0.363	ID	0.225	0.236	0.235	0.281	0.285	0.214	0.21	0.21	0.166	0.194	0.188	0.242
EgrARF5	0.247	0.247	0.252	0.197	0.225	ID	0.371	0.379	0.236	0.242	0.185	0.18	0.192	0.15	0.32	0.328	0.203
EgrARF6A	0.261	0.261	0.265	0.196	0.236	0.371	ID	0.678	0.263	0.265	0.191	0.187	0.193	0.155	0.35	0.337	0.217
EgrARF6B	0.267	0.262	0.266	0.2	0.235	0.379	0.678	ID	0.268	0.266	0.194	0.197	0.203	0.162	0.361	0.358	0.224
EgrARF9A	0.503	0.372	0.387	0.237	0.281	0.236	0.263	0.268	ID	0.668	0.238	0.236	0.239	0.198	0.21	0.208	0.321
EgrARF9B	0.471	0.371	0.388	0.241	0.285	0.242	0.265	0.266	0.668	ID	0.224	0.223	0.228	0.189	0.207	0.203	0.301
EgrARF10	0.237	0.202	0.211	0.21	0.214	0.185	0.191	0.194	0.238	0.224	ID	0.564	0.577	0.302	0.166	0.166	0.202
EgrARF16A	0.245	0.204	0.205	0.207	0.21	0.18	0.187	0.197	0.236	0.223	0.564	ID	0.556	0.301	0.162	0.156	0.204
EgrARF16B	0.233	0.206	0.204	0.202	0.21	0.192	0.193	0.203	0.239	0.228	0.577	0.556	ID	0.311	0.164	0.165	0.214
EgrARF17	0.2	0.167	0.166	0.18	0.166	0.15	0.155	0.162	0.198	0.189	0.302	0.301	0.311	ID	0.125	0.135	0.199
EgrARF19A	0.208	0.215	0.218	0.165	0.194	0.32	0.35	0.361	0.21	0.207	0.166	0.162	0.164	0.125	ID	0.476	0.159
EgrARF19B	0.206	0.218	0.218	0.164	0.188	0.328	0.337	0.358	0.208	0.203	0.166	0.156	0.165	0.135	0.476	ID	0.163
EgrARF24	0.329	0.296	0.317	0.226	0.242	0.203	0.217	0.224	0.321	0.301	0.202	0.204	0.214	0.199	0.159	0.163	ID
