# Supplementary material for: Genome-Wide Characterization and Expression Profiling of the AUXIN RESPONSE FACTOR (ARF) Gene Family in Eucalyptus grandis
Source: PLoS One. 2014 Sep 30;9(9):e108906. doi: 10.1371/journal.pone.0108906 (PMC4182523; doi:10.1371/journal.pone.0108906)
Supplement: Table S4 — Comparison of the number of alternative transcripts predicted in phytozome for E. grandis to those found in a large compendium of transcriptomic data from in E. globulus . (PDF) [file pone.0108906.s014.pdf]

Table S4 Comparison of the number of alternative transcripts predicted in phytozome for *E. grandis* to those found in *E. globulus* in a large compendium transcriptomic data

Gene name	*E. grandis*	*E. globulus*
EgrARF1	4	6
EgrARF2A	5	11
EgrARF2B	2	3
EgrARF3	2	5
EgrARF4	3	4
EgrARF5	1	4
EgrARF6A	2	6
EgrARF6B	3	4
EgrARF9A	1	2
EgrARF9B	2	3
EgrARF10	1	1
EgrARF16A	1	1
EgrARF16B	1	3
EgrARF17	1	4
EgrARF19A	2	7
EgrARF19B	2	6
EgrARF24	1	1

These numbers include the primary transcripts

Material and Methods for Alternative slipicing validation

TopHat and Cufflink Suite (Trapnell et al. 2012), currently used for differential gene and transcript expression analysis of RNA-seq experiments, were used to validate the *Eucalyptus ARF* genes (*E. grandis* genome v162 annotation).

Ten developing xylem (DX) RNA-Seq libraries were produced and sequenced FASTERIS SA (Genève, CH; www.fasteris.com). RNA-Seq libraries were prepared from ten equimolar pools of high quality individual total RNA: a) four seasonal libraries produced from total RNA extracted from DX samples collected in 2008 on three ramets of non-related tree genotypes at Herdade do Zambujal, Pegões (Portugal) [February 26 th (Feb), May 23rd (May), September 5th (Sep) and December 3rd (Dec)] (Carocha et al., unpublished); b) four pulp yield pools (five trees by pool) obtained by pooling the total RNA from samples

collected at Carregal Fundeiro, Abrantes (Portugal), from 10 trees with very constrasting pulp yields in the coded AxB mapping population, and 10 trees with very constrasting pulp yields in a natural variation panel; c) two libraries from samples collected from an adult tree and a juvenile one, (same genotype) at Herdade do Zambujal, Pegões (Portugal). Sampling procedures and RNA extraction were described, respectively in Paux et al. (2004) and Cassan- Wang et al. (2012). All samples were kindly provided by RAIZ Institute (Portugal).

The TruSeq™ SBSv5 sequencing kit (Illumina) was used for library sequencing, using the Illumina Hi-Seq 2000 instrument, on a multiplex runs with 1x100nt+7(index) cycles. For each RNA-Seq library, adaptors removal and a successive quality and contaminants (ribosomal sequences) filters were applied. The resulting filtered high quality reads were then mapped to the *E. grandis* genome sequence v162 using TopHat v1.3.1. Cufflinks v1.1.0 was used to determine the potential coding regions and their intron-exon structures. Finally, Cuffcompare v1.1.0 were then used to compare the assembled multiple libraries Cufflinks transcripts to the *E. grandis* v162 gene annotations.

The sequences of the Illumina reads from RNA Seq used to predict the *E. globulus* alternative transcripts are provided in FastaQ format in supplementary File S1.

REF:

Cole Trapnell, Adam Roberts, Loyal Goff, Geo Pertea, Daehwan Kim, David R Kelley, Harold Pimentel, Steven L Salzberg, John L Rinn & Lior Pachter (2012) Differential gene and transcript expression analysis of RNA-seq experiments with TopHat and Cufflinks. Nature Protocols 7: 562–578
