# Supplementary material for: Genome-Wide Characterization and Expression Profiling of the AUXIN RESPONSE FACTOR (ARF) Gene Family in Eucalyptus grandis
Source: PLoS One. 2014 Sep 30;9(9):e108906. doi: 10.1371/journal.pone.0108906 (PMC4182523; doi:10.1371/journal.pone.0108906)
Supplement: Table S5 — Comparison of the number of EgrARF24 putative orthologs in other species. (PDF) [file pone.0108906.s015.pdf]

Table S5. The number of *EgrARF24* orthologs in other species

Species	Number of orthologs*
Brassica rapa	0
Gossypium raimondii	2
Malus domestica	2
Medicago truncatula	0
Citrus sinensis	1
Citrus clementina	1
Prunus persica	1
Solanum lycopersicum	0
Manihot esculenta	0
Ricinus communis	0
Linum usitatissimum	0
Phaseolus vulgaris	1
Glycine max	2
Cucumis sativus	0
Fragaria vesca	1
Arabidopsis lyrata	0
Capsella rubella	0
Thellungiella halophila	0
Carica papaya	1
Theobroma cacao	1
Solanum tuberosum	0
Mimulus guttatus v1.1	0
Aquilegia coerulea	1
Sorghum bicolor	0
Setaria italica	0
Panicum virgatum	0
Brachypodium distachyon	0
Selaginella moellendorffii	0
Populus trichocarpa	2
Arabidopsis thaliana	0
Zea mays	0
Oryza sativa	0
Vitis vinifera	1
Eucalyptus grandis	1

**The number of EgrARF24 orthologs in other species are counted in phytozome by using "Filter homologs" with a cut off E value 1.0E-50*
