# Supplementary material for: Genome-Wide Characterization and Expression Profiling of the AUXIN RESPONSE FACTOR (ARF) Gene Family in Eucalyptus grandis
Source: PLoS One. 2014 Sep 30;9(9):e108906. doi: 10.1371/journal.pone.0108906 (PMC4182523; doi:10.1371/journal.pone.0108906)
Supplement: Table S6 — Potential small RNAs targeting EgrARF genes. (PDF) [file pone.0108906.s016.pdf]

Table S6. Potential small RNAs which target for *EgrARF* genes

Small RNA gene	Small RNA sequence (5'-3')	Length	Genomic location	Predicted small RNA gene sequences (5'-3')
MIR160				
EgrMIR160a	UGCCUGGCUCCCUGUAUGCCA	21	scaffold_4:29,715,926..29,716,011	TGCCTGGCTCCCTGTATGCCACAAACATATTCCAACCTTTCATCTTAGGTTGGCCATTACGTTGGTGGCGTACCAGGAGCCAAGCC
EgrMIR160b	UGCCUGGCUCCCUGUAUGCCA	21	scaffold_7:46392610..46392691	TGCTTGGCTCCTCATACGCCATCCGCGGAGACCATCGATTCTCCGATGCGCTCTCTGCACATGGCATACAGGGAGCCAGGCA
EgrMIR160c	UGCCUGGCUCCCUGUAUGCCA	21	scaffold_10:12063249..12063328	TGCATGGCTCCTCATACGCCATACACACAGGCCATCGGTTCTCCGGTGGGCTACGCAAATGGCATACAGGGAGCCAGGCA
MIR167				
EgrMIR167a	UGAAGCUGCCAGCAUGAUCU	20	scaffold_8:49,907,076..49,907,178	GAGATGCCAGCAACAGGTGAAGATGCCACATGATCTGATCCATCCTCAAGTCAAAGAGGGAGGTTGAGATCATGCTGGCAGCTTCAACCGATGCTGGTCTCTC
EgrMIR167b	UGAAGCUGCCAGCAUGAUCU	20	scaffold_8:62,733,419..62,733,519	TCATGCTTCACAGCAACAGTTGAAACTGCCAGATGATCTGACAGCTTCTTCTATTGAAGGCTCAGATCATGCTGGCAGCTTCAGCTATTAGTGGTGCATGA
EgrMIR167c	UGAAGCUGCCAGCAUGAUCUA	21	scaffold_11:42,258,705..42,258,841	AAGGGAACGAGTGAAGCTGCCAGCATGATCTAGCTCCGGCTAACTTGAAACTTCAACCGAAAAAAAAAAAAAAACAAGAAAAGAAGAAGTGGTTAACCCTAGCTAGGTCATGCTGCGACAGCCTCACT
EgrTAS3	UUCUUGACCUUGUAAGACCUU	21	scaffold_8:58712106..58712287	TTCTTGACCTTGTAAGACCTTTTCTTGACCTTGTAAGACCCCCCTCCGTTCCCGCCCCGTCACTTCTCCTTCCGGCCCCGGGCTCTGAATCGCGAGTGAGCGATGTAGAGTACGGTCGCCTGCCTTCCCCTTTCCCACCCAACTCATCTTCTCCTTCCTTGTCTATCCCTCCTGAGCTACTC
