# Supplementary material for: Genome-Wide Characterization and Expression Profiling of the AUXIN RESPONSE FACTOR (ARF) Gene Family in Eucalyptus grandis
Source: PLoS One. 2014 Sep 30;9(9):e108906. doi: 10.1371/journal.pone.0108906 (PMC4182523; doi:10.1371/journal.pone.0108906)
Supplement: Table S7 — Small RNAs target site prediction in EgrARF genes. (PDF) [file pone.0108906.s017.pdf]

Table S7. Small RNAs target site prediction of *EgrARF* genes

miRNA Acc.	Target Acc.	Expectation[a] (E)	Target Accessibility[b] (UPE)	Alignment	Inhibition[c]	Multiplicity[d]
UGCCUGGCUCCCUGUAUGCCA<br>EgrmiR160	Eucgr.J00923.1<br>EgrARF10	0	20.2	miRNA 20 CCGUAUGUCCCUCGGUCCGU 1<br>::::::::::::::::::::<br>Target 1804 GGCAUACAGGGAGCCAGGCA 1823	Cleavage	1
UGCCUGGCUCCCUGUAUGCCA<br>EgrmiR160	Eucgr.G02838.1<br>EgrARF16A	0	20.2	miRNA 20 CCGUAUGUCCCUCGGUCCGU 1<br>::::::::::::::::::::<br>Target 1435 GGCAUACAGGGAGCCAGGCA 1454	Cleavage	1
UGCCUGGCUCCCUGUAUGCCA<br>EgrmiR160	Eucgr.K01240.1<br>EgrARF16B	1	22.4	miRNA 21 ACCGUAUGUCCCUCGGUCCGU 1<br>:::.::.::::::::::::::<br>Target 2095 UGGUAUGCAGGGAGCCAGGCA 2115	Cleavage	1
UGCCUGGCUCCCUGUAUGCCA<br>EgrmiR160	Eucgr.F04380.1<br>EgrARF17	0.5	18.0	miRNA 21 ACCGUAUGUCCCUCGGUCCGU 1<br>::::::.::::::::::::::<br>Target 1218 UGGCAUGCAGGGAGCCAGGCA 1238	Cleavage	1
UGAAGCUGCCAGCAUGAUCUA<br>EgrmiR167	Eucgr.D00264.1<br>EgrARF6A	3.5	22.2	miRNA 20 UCUAGUACGACCGUCGAAGU 1<br>:::::: :::::::::::::<br>Target 2416 AGAUCAGGCUGGCAGCUUGU 2435	Cleavage	1
UGAAGCUGCCAGCAUGAUCUA<br>EgrmiR167	Eucgr.A02065.1<br>EgrARF6B	3.5	23.4	miRNA 20 UCUAGUACGACCGUCGAAGU 1<br>:::::: :::::::::::::<br>Target 3043 AGAUCAGGCUGGCAGCUUGU 3062	Cleavage	1
UUCUUGACCUUGUAAGACCUU<br>EgrTAS3	Eucgr.K02197.1<br>EgrARF2A	3.5	17.3	miRNA 21 UUCCAGAAUGUUCCAGUUCUU 1<br>:.: ::::.::::::: ::::<br>Target 1249 AGGAUCUUGCAAGGUCCAGAA 1269	Cleavage	1
UUCUUGACCUUGUAAGACCUU<br>EgrTAS3	Eucgr.B03551.1<br>EgrARF2B	1.5	18.5	miRNA 21 UUCCAGAAUGUUCCAGUUCUU 1<br>:::: :::.::::::::::::<br>Target 1641 AAGGCCUUGCAAGGUCAAGAA 1661	Cleavage	1
UUCUUGACCUUGUAAGACCUU<br>EgrTAS3	Eucgr.D00588.1<br>EgrARF3	0.5	20.5	miRNA 21 UUCCAGAAUGUUCCAGUUCUU 1<br>::::::::.::::::::::::<br>Target 1641 AAGGUCUUGCAAGGUCAAGAA 1661	Cleavage	1
UUCUUGACCUUGUAAGACCUU<br>EgrTAS3	Eucgr.B02480.1<br>EgrARF4	0.5	24.1	miRNA 21 UUCCAGAAUGUUCCAGUUCUU 1<br>::::::::.::::::::::::<br>Target 1631 AAGGUCUUGCAAGGUCAAGAA 1651	Cleavage	1

[a]The complementarity score between small RNA and and their target transcript, if its score is greater than 4 it will be discarded

[b]The energy required to open (unpair) secondary structure around target site on target mRNA, The less energy means the more possibility that small

[C]The principle of the miRNA function, cleave mRNA or inhibits the translation of target genes

[d]The number of small RNA target sites on each mRNA
